# Selective monoformylation of naphthalene-fused propellanes for methylene-alternating copolymers

**DOI:** 10.3762/bjoc.21.95

**Published:** 2025-06-18

**Authors:** Kenichi Kato, Tatsuki Hiroi, Seina Okada, Shunsuke Ohtani, Tomoki Ogoshi

**Affiliations:** 1 Department of Synthetic Chemistry and Biological Chemistry, Graduate School of Engineering, Kyoto University, Katsura, Nishikyo-ku, Kyoto, 615-8510, Japan; 2 WPI Nano Life Science Institute, Kanazawa University, Kakuma-machi, Kanazawa, 920-1192, Japan

**Keywords:** alternating copolymer, building block, formylation, gas adsorption, propellane

## Abstract

Development of three-dimensional (3D) building blocks is a key to change tight molecular assemblies of rigid π-conjugated planes into organic functional materials endowed with molecular-size cavities. To increase the diversity of available 3D building blocks, we herein report electrophilic formylation of naphthalene-fused [3.3.3]- and [4.3.3]propellanes as the first selective single-point functionalization by virtue of through-space electronic communications between the naphthalene units. The propellane skeletons have well-defined 3D structures and moderate flexibility at the same time. Therefore, the monoformyl products are good precursors for soft materials which show molecular-size cavities and require desymmetrized building blocks. As a proof of concept, methylene-alternating copolymers were prepared by reduction to corresponding alcohols followed by acid-mediated condensation. The linear copolymers show good solubility and carbon dioxide adsorption.

## Introduction

Combination of sp^2^- and sp^3^-hybridized atoms in core π-skeletons [1–3] is a key to go beyond common organic functional materials composed of rigid π-conjugated planes and flexible peripheral substituents. Because larger π-conjugated planes mostly display low solubility and dense packing due to the π–π stacking and CH–π interactions, surrounding alkyl and other flexible moieties are widely adopted to improve the solubility and modulate the molecular assemblies [4–9]. By contrast, the presence of sp^3^-hybridized atoms in core π-skeletons can lead to three-dimensional (3D) structures with appropriate rigidity, thereby giving macrocyclic arenes [10–11], molecule-based cages and frameworks [12–19], polymers of intrinsic microporosity [20–24], and so forth. Characteristically, they possess molecular-size cavities, which contribute to intricate molecular recognition [25], confined spaces for reactions [26], and small-molecule storage and transport [27–29]. Further progress in such unique organic materials largely depends on the exploitation of 3D π-building blocks. However, the variety of building blocks are limited to a few families such as tetraphenylmethane and triptycene [30–39].

**Figure 1:**
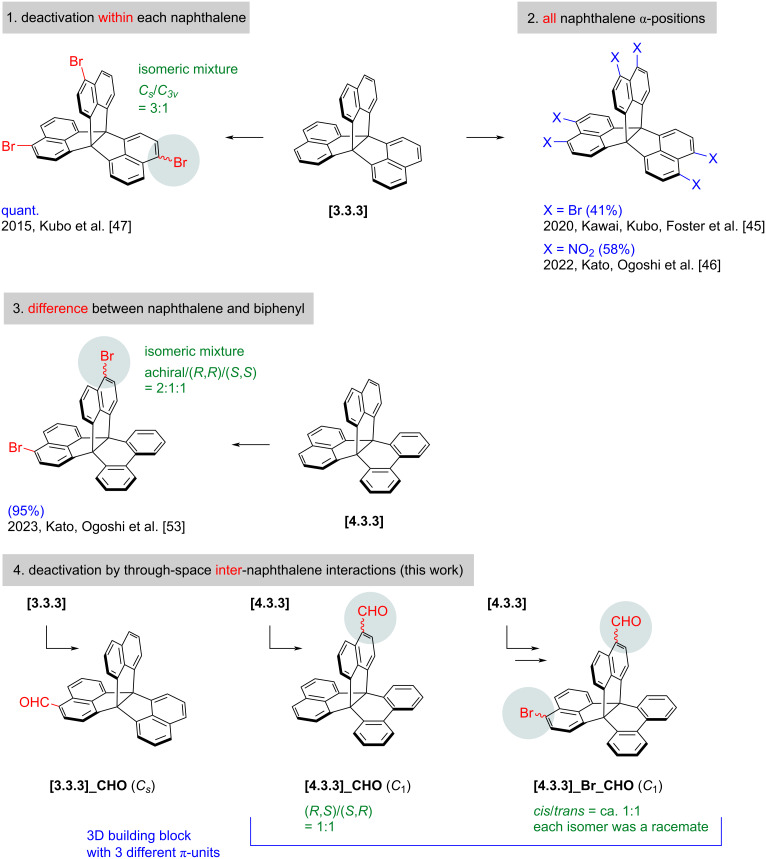
Chemical structures of fully π-fused propellanes and their typical reaction patterns toward electrophilic functionalization.

Widespread use of 3D π-skeletons requires not only efficient construction of the skeletons but also functionalization with precise control of substitution numbers and positions. Along this line, fully π-fused [4.4.4]- and [3.3.3]propellanes [40–43] were able to be brominated and nitrated at six positions while retaining molecular symmetry (Figure 1) [44–46]. One functional group was selectively introduced to each naphthalene ring of fully π-fused [4.3.3]- and [3.3.3]propellane, **[4.3.3]** and **[3.3.3]**, respectively [47–55]. In this work, we report the introduction of a single functional group to a whole skeleton of **[4.3.3]** and **[3.3.3]**, using formylation [55–56]. The reaction is electrophilic, and the substrates are effectively deactivated toward further reactions upon introduction of an electron-withdrawing formyl group because of through-space electronic interactions between the naphthalene units. The monoformyl products are reduced to corresponding alcohols, which are then reacted under Friedel–Crafts conditions. Amorphous methylene-alternating copolymers are obtained without particular macrocyclic oligomers. Due to the 3D components, the linear copolymers display good solubility in CHCl_3_ and THF and adsorption properties for CO_2_ gas.

## Results and Discussion

### Selective monoformylation

Initially, we tried introducing formyl groups into a fully π-fused [4.3.3]propellane via organometal species, which had been effective for functional π-extended systems [57–60]. This scheme also enables control of the number of formyl groups by starting materials and reagents. Brominated [4.3.3]propellane was reacted with *n*-BuLi or iPrMgCl·LiCl to generate an organometal species, which was quenched with *N*,*N*-dimethylformamide (DMF) as an electrophile (Table S201 in Supporting Information File 1). Despite several trials, the reactions led to complicated mixtures owing to decomposition and debromination or predominant recovery of the starting material, respectively.

Then, we turned our attention to electrophilic formylation. Vilsmeier–Haack [61] and Duff [62] reactions led to recovery of starting material or a complicated mixture probably owing to the modestly electron-rich and sterically demanding naphthalene α-positions (Table S202, entries 1–3, Supporting Information File 1). By contrast, a combination of dichloromethyl methyl ether and TiCl_4_ (Rieche reaction) [55–56,63–66] yielded the monoformyl product **[4.3.3]_CHO**, in a selective manner (Table 1, entry 1). To suppress decomposition in the overnight reaction at room temperature, the reaction time was reduced to 1.5 h, which afforded **[4.3.3]_CHO** in an isolated yield of 80% (Table 1, entry 2). The same protocol was successfully applicable to pristine π-fused [3.3.3]propellane **[3.3.3]**, giving **[3.3.3]_CHO** selectively in 67% yield (Table 1, entry 3).

**Table 1:** Formylation of naphthalene-fused propellanes.

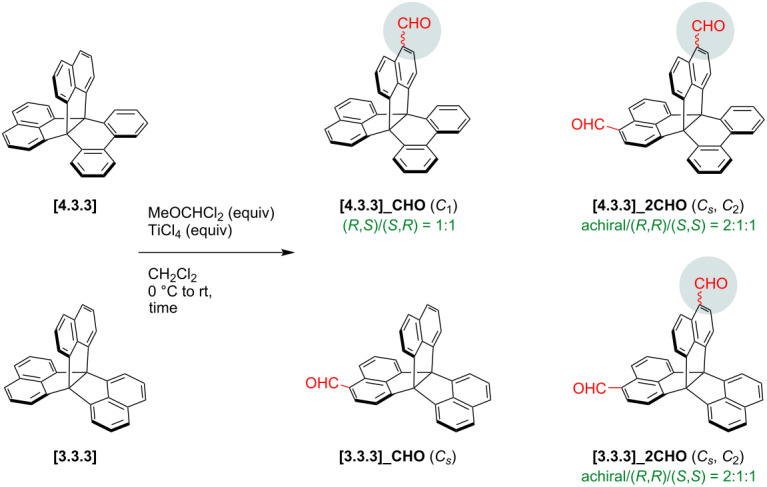				

entry	substrate	equiv	time	results

1	**[4.3.3]**	1.25	29 h	**[4.3.3]_CHO** (48%)
2	**[4.3.3]**	1.2	1.5 h	**[4.3.3]_CHO** (80%), **[4.3.3]** (18%)
3	**[3.3.3]**	1.2	1.5 h	**[3.3.3]_CHO** (67%), **[3.3.3]** (12%), **[3.3.3]_2CHO** (5.1%)
4	**[3.3.3]**	2.4	1.5 h	**[3.3.3]_2CHO** (25%), **[3.3.3]_CHO** (61%)
5	**[4.3.3]**	2.4	1.5 h	**[4.3.3]_2CHO** (1.8%), **[4.3.3]_CHO** (56%)
6	**[4.3.3]**	2.4	18 h	**[4.3.3]_2CHO** (9.9%), **[4.3.3]_CHO** (33%)
7^a^	**[4.3.3]_Br**	1.2	18 h	**[4.3.3]_Br_CHO** (39% in 2 steps from **[4.3.3]**)

^a^Substrate was a 1:2:1 mixture of **[4.3.3]**, **[4.3.3]_Br**, and **[4.3.3]_2Br**, obtained by bromination of **[4.3.3]** with 1.03 equiv of Br_2_ (Figure S201 in Supporting Information File 1) [53].

In electrophilic aromatic substitutions, multifold reactions are possible, and the number of substitution is sometimes difficult to control by tuning reaction temperature and time. Indeed, bromination of **[3.3.3]** and **[4.3.3]** was reported as three/six- and two-fold reactions, respectively [45,47,53]. If the amount of bromine was limited, the resulting nearly random mixtures of brominated compounds would be practically impossible to separate by chromatography on silica gel because of their low polarity and poor solubility in *n*-hexane. On the other hand, nitration of **[3.3.3]** gave solely the six-fold nitrated product due to low solubility of the starting material [46]. The current reaction is the first practical method for the selective monofunctionalization of **[3.3.3]** and **[4.3.3]**, to the best of our knowledge. It is also noteworthy that this reaction further desymmetrized the [4.3.3]propellane skeleton of **[4.3.3]** into a 3D building block bearing three different fused π-units.

### Diformylation and computed electronic structures

In the formylation of **[3.3.3]**, diformylated product **[3.3.3]_2CHO** was obtained in 5.1% yield. In expectation of successful multifold formylation, the equivalents of dichloromethyl methyl ether and TiCl_4_ were doubled (Table 1, entry 4). The yield of **[3.3.3]_2CHO** modestly increased to 25% with a slight decrease in the yield of **[3.3.3]_CHO** (61%). Although further increase of the equivalents and prolonged reaction time may potentially provide better results for **[3.3.3]_2CHO**, we gave up such attempts because of the competing decomposition in these strongly acidic conditions. In the case of **[4.3.3]**, diformylation gave only 1.8% of **[4.3.3]_2CHO** after 1.5 h (Table 1, entry 5), which was consistent with the absence of **[4.3.3]_2CHO** in monoformylation. Due to the low reactivity, the reaction time was elongated to 18 h (Table 1, entry 6). The yield of **[4.3.3]_2CHO** was improved to 9.9%, whereas the yield of **[4.3.3]_CHO** decreased to 33% owing to competing decomposition. As a substrate, a mixture obtained by monobromination of **[4.3.3]** could be used, giving difunctional building block **[4.3.3]_Br_CHO** in 39% yield (Table 1, entry 7). The reaction was highly successful because the bromination gave nearly random 1:2:1 mixtures of **[4.3.3]**, **[4.3.3]_Br** and **[4.3.3]_2Br**.

To gain insight into the different reactivity between **[3.3.3]** and **[4.3.3]**, theoretical calculations were performed at the ωB97X-D/6-31G(d,p) level of theory (Figures S901–S903 in Supporting Information File 1). Although distribution of the highest occupied molecular orbitals (HOMOs) was similarly delocalized to multiple naphthalene units, the energy for **[3.3.3]** (−7.23 eV) was higher than that of **[4.3.3]** (−7.32 eV). Upon formylation, the HOMO energies of **[3.3.3]** and **[4.3.3]** were stabilized to −7.44 eV and −7.55 eV by 0.21 eV and 0.23 eV, respectively. These values correlated well with the observed reactivities and selectivity.

### Attempted macrocyclization leading to linear polymers

**Figure 2:**
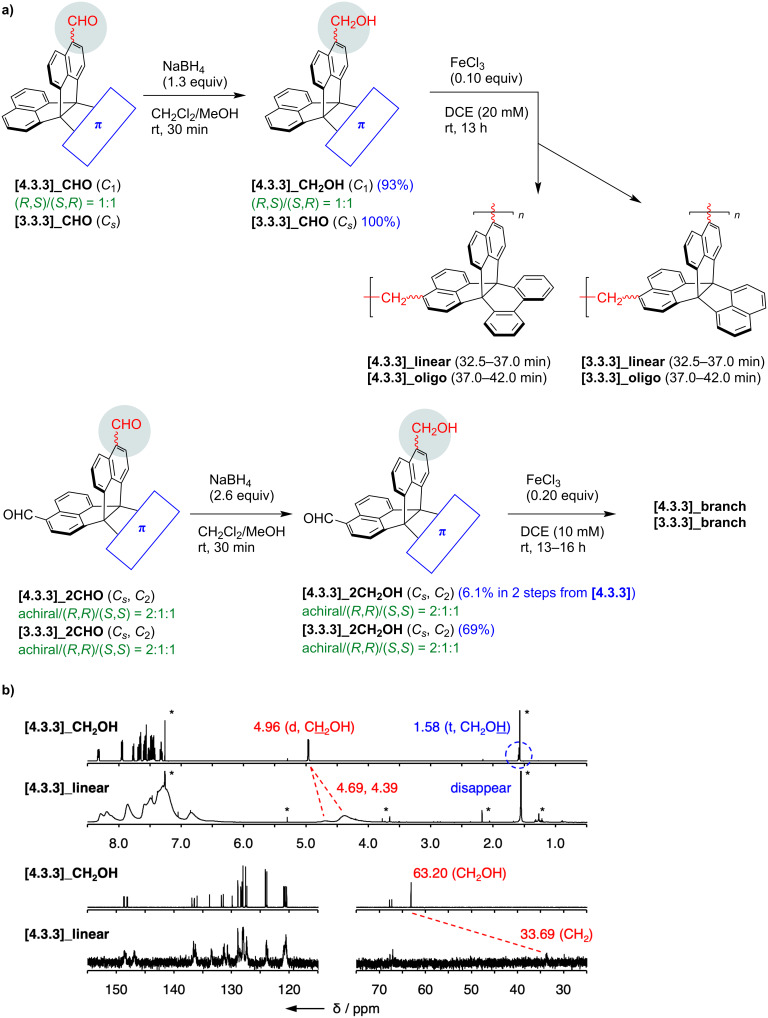
a) Synthesis of methylene-alternating copolymers of fully π-fused propellanes. DCE, 1,2-dichloroethane. b) ^1^H NMR (500 MHz, top) and ^13^C (126 MHz, bottom) NMR spectra of **[4.3.3]_CH_2_OH** and **[4.3.3]_linear** in CDCl_3_ at room temperature.

Formyl groups have diverse reactivities and enable facile condensation, dynamic covalent chemistry, and so on. In this work, we tried the synthesis of cyclic oligomers composed of naphthalene-fused propellanes simply by reduction into the corresponding alcohols followed by acid-mediated Friedel–Crafts-type reactions (Figure 2a and Figure S201 in Supporting Information File 1) [67–68]. Reduction by NaBH_4_ proceeded well for both monoaldehydes **[4.3.3]_CHO** and **[3.3.3]_CHO** resulting in over 90% yield. Alcohol products, **[4.3.3]_CH_2_OH** and **[3.3.3]_CH_2_OH**, were then tested in acidic conditions using anhydrous FeCl_3_ as a Lewis acid. After the reactions, the alcohol proton signals at 1.54–1.58 ppm disappeared in the ^1^H NMR spectra, and aliphatic carbon ones at 63.1–63.2 ppm were largely up-field-shifted to ca. 34 ppm in the ^13^C NMR spectra due to conversion into methylene groups (Figure 2b and Figures S315 and S316 in Supporting Information File 1). However, all ^1^H NMR signals were broad, and gel permeation chromatography (GPC) charts indicated broad patterns due to multiple products with varying molecular weights. These results implied that formation of well-defined cyclic oligomers was quite limited. To increase the well-defined species, **[4.3.3]_CH_2_OH** was separated into two enantiopure fractions (Supporting Information File 1, Figure S505), one of which was used for the acid-mediated reaction. Despite the stereocontrolled substrate, the resonances in the ^1^H NMR spectrum of the product remained broad. Therefore, we concluded that these systems were difficult to give specific macrocyclic oligomers but instead provided linear polymers composed of fully π-fused propellanes.

After the reactions, each product was separated into oligomer and linear polymer by preparative GPC using CHCl_3_ as eluent. According to analytical GPC in THF (Figures S501–504 in Supporting Information File 1), oligomer fractions were mainly composed of tetramer for **[3.3.3]_oligo** and dimer and trimer for **[4.3.3]_oligo**. Fractions of linear polymers indicated peak top molecular weights at around octamer for **[3.3.3]_linear** and pentamer and hexamer for **[4.3.3]_linear** (see also Table 2). In analogy with monoaldehydes, dialdehydes were reduced to dialcohols, **[3.3.3]_2CH_2_OH** and **[4.3.3]_2CH_2_OH**, and polymerized in acidic conditions (Figure 2a). Insoluble solids, **[3.3.3]_branch** and **[4.3.3]_branch**, were obtained due to formation of bonding networks and washed repeatedly with CH_2_Cl_2_, H_2_O, and acetone.

**Table 2:** Properties of methylene-alternating copolymers.^a^

	*M*_n_	*M*_w_	*M*_w_ */M*_n_	*T*_90_ [°C]	CY [wt %]	*V* (CO_2_) [cm^3^ g^−1^]	*S*_BET_ [m^2^ g^−1^]

**[3.3.3]_oligo**	–	–	–	532	76	22	–
**[3.3.3]_linear**	3.29 × 10^3^	3.79 × 10^3^	1.15	528	68	24	–
**[3.3.3]_branch**	–	–	–	415	64	18	61
**[4.3.3]_oligo**	–	–	–	468	47	15	–
**[4.3.3]_linear**	2.69 × 10^3^	3.16 × 10^3^	1.17	491	46	15	–
**[4.3.3]_branch**	–	–	–	543	75	29	323

^a^*M*_n_, number-average molar mass; *M*_w_, mass-average molar mass; *T*_90_, temperature at which weight loss reaches 10%; CY, carbonization yield; *V* (CO_2_), CO_2_ uptake (STP) at 90 kPa; *S*_BET_, BET surface area.

### Characterization of methylene-alternating copolymers

The thermal stability of the oligomers and polymers were evaluated with thermogravimetric analysis (TGA) (Figure S703 in Supporting Information File 1). Temperatures at which weight loss reached 10% (*T*_90_) were 468–491 °C and carbonization yields at 900 °C (CY) were ca. 46 wt % for **[4.3.3]_oligo** and **[4.3.3]_linear**. *T*_90_ and CY of **[4.3.3]_branch** showed higher values of 543 °C and 75 wt % probably owing to the network structure. By contrast, soluble **[3.3.3]_oligo** and **[3.3.3]_linear** had relatively high *T*_90_ of 528–532 °C and CY of 68–76 wt %. The high values were ascribed to two unsubstituted naphthalene rings in precursor **[3.3.3]_CH_2_OH**, which caused facile branching in the reaction or heating process. *T*_90_ and CY of **[3.3.3]_branch** (415 °C and 64 wt %) were lower than those of **[3.3.3]_linear** because of two-step decay profile (Figure S703a in Supporting Information File 1).

All the samples showed broad powder X-ray diffraction (PXRD) patterns with unclear peaks at around 2θ = 11° and 20° (Supporting Information File 1, Figure S601) and continuous curves in differential scanning calorimetry (DSC) between −70 and 300 °C (Figures S701 and S702, Supporting Information File 1). The results indicated that the polymers were amorphous while giving relatively high thermal stability toward phase transition and decomposition.

**Figure 3:**
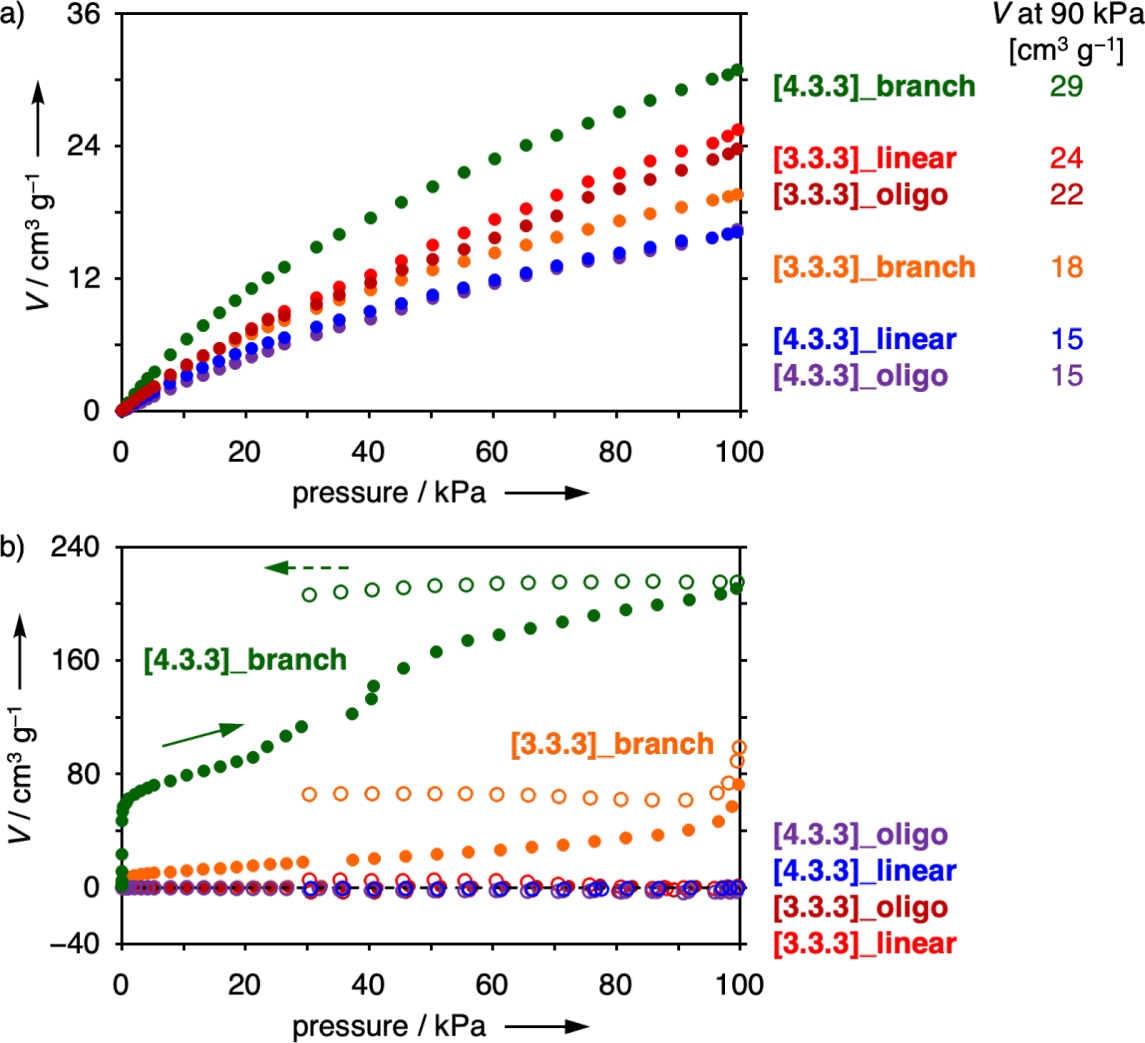
Gas adsorption (filled circles) and desorption (open circles) isotherms of **[3.3.3]_oligo** (dark red), **[3.3.3]_linear** (red), **[3.3.3]_branch** (orange), **[4.3.3]_oligo** (purple), **[4.3.3]_linear** (blue), and **[4.3.3]_branch** (green). a) CO_2_ at 298 K and b) N_2_ at 77 K.

Then, gas adsorption properties [46,69–72] were evaluated after the samples were activated in vacuo at 120 °C (Figure 3 and Figures S801 and S802 in Supporting Information File 1). Their chemical structures did not necessarily contain branched or ladder-type connections, but all of them displayed CO_2_ adsorption properties at 298 K probably due to the 3D components. The uptake values at standard temperature and pressure (STP) were 15–29 cm^3^·g^−1^ at 90 kPa. In this series, a sample with higher *T*_90_ and CY values tended to exhibit a higher adsorption capacity for CO_2_. By contrast, the linear oligomers and polymers did not adsorb N_2_ gas at 77 K. The adsorption isotherms of branched polymers did not have major IUPAC type-I contributions either, indicating the absence of micropores suitable for N_2_ adsorption. The curve for **[3.3.3]_branch** looked like type-II, and that of **[4.3.3]_branch** showed multistep uptake. In the desorption step, both samples retained most of the adsorbed N_2_ molecules even at 30 kPa. These observations and slow equilibrium in the adsorption processes suggested that presence of narrow connections between molecular-size cavities disturbed smooth N_2_ adsorption and desorption. Molecular design for uniform microporosity and efficient polymerization is a next challenge.

## Conclusion

In this work, we developed formylation on a naphthalene ring in [3.3.3]- and [4.3.3]-type fully π-fused propellanes. High selectivity was achieved for monoformylation on a naphthalene ring. It was reported that bromination proceeded in three- or six-fold manners for a [3.3.3]propellane [45,47–52], and in two-fold one for a [4.3.3]propellane [53–54]. Nitration of the [3.3.3]propellane also yielded an exclusive six-fold product [46]. The current formylation is valuable as the first reliable method for monofunctionalization of naphthalene-fused propellanes without giving inseparable mixtures with multi-functionalized products. Due to the wide reactivities of the formyl group, the monoformyl propellanes would promote new research domains on non-branched linear polymers, macrocyclic compounds, and molecular assemblies that incorporate propellanes as key 3D components. As a proof of concept, the formylated products were reduced to the corresponding alcohols and polymerized in acidic conditions. Although the degrees of polymerization were not high, the methylene-alternating copolymers displayed gas adsorption properties. Further studies are underway towards novel functional materials containing fully π-fused propellanes as flexible 3D building blocks.

## Supporting Information

Supporting information includes general information, synthetic procedures and compound data, NMR and MS spectra, HPLC charts, and results of PXRD, DSC, TGA, gas adsorption, and theoretical calculations.

File 1 Experimental.

## Data Availability

The data that supports the findings of this study is available in the supporting information of this article. Further data generated and analyzed during this study is available from the corresponding author upon reasonable request.
