# Supplementary material for: Selective monoformylation of naphthalene-fused propellanes for methylene-alternating copolymers
Source: Beilstein J Org Chem. 2025 Jun 18;21:1183–91. doi: 10.3762/bjoc.21.95 (PMC12207256; doi:10.3762/bjoc.21.95)
Supplement: File 1 — Experimental. [file Beilstein_J_Org_Chem-21-1183-s001.pdf]

BEILSTEIN JOURNAL OF ORGANIC CHEMISTRY

Supporting Information

for

Selective monoformylation of naphthalene-fused propellanes for methylene-alternating copolymers

Kenichi Kato, Tatsuki Hiroi, Seina Okada, Shunsuke Ohtani and Tomoki Ogoshi

Beilstein J. Org. Chem. **2025,** *21,* 1183–1191. doi:10.3762/bjoc.21.95

Experimental

License and Terms: This is a supporting information file under the terms of the Creative Commons Attribution License (https://creativecommons.org/licenses/by/4.0). Please note that the reuse, redistribution and reproduction in particular requires that the author(s) and source are credited and that individual graphics may be subject to special legal provisions.
The license is subject to the *Beilstein Journal of Organic Chemistry* terms and conditions: (https://www.beilstein-journals.org/bjoc/terms)

Table of contents

1. General information
2. Synthetic procedures and compound data
3. ^{1}H and ^{13}C NMR spectra
4. HR APCI–orbitrap-MS, ESI–orbitrap-MS, and EI–selector-MS
5. HPLC charts
6. X-Ray diffraction data
7. DSC and TGA measurement
8. Gas adsorption measurement
9. Theoretical calculations
10. References

1. General information

Materials in synthesis

All reagents and solvents were of commercial reagent grade and were used without further purification except where noted. Dibrominated (dibenzo)(dinaphtho)[4.3.3]propellane **[4.3.3]_2Br**[S1] (Table S201), (dibenzo)(dinaphtho)[4.3.3]propellane **[4.3.3]**,[S1,S2] and trinaphtho[3.3.3]propellane **[3.3.3]**[S2,S3] (Scheme S201) were prepared according to the reported methods. Dehydrated stabilizer-free tetrahydrofuran (THF, Super plus) and dehydrated CH_2Cl_2 (Super[2]) and dehydrated *N*,*N*-dimethylformamide (DMF, Super) were purchased from Kanto Chemical Co., Inc. Chromatography-grade activated aluminum oxide was purchased from FUJIFILM Wako Pure Chemical Industry, Ltd. Dry 1,2-dichloroethane was passed through a short aluminum oxide pad and Wakosil 60 and degassed by N_2 bubbling before use. Deionized water was obtained from a Merck Elix-Essential-3 instrument with a Progard TS2 Pretreatment Pack. Thin-layer chromatography (TLC) analyses were performed on commercial aluminum plates bearing a 0.25 mm layer of Merck silica gel 60 F_{254}. Preparative silica gel and chromatography was performed on Wakosil 60 or Wakogel C-400HG. Gel permeation chromatography (GPC) was performed on a Japan Analytical Industry LaboACE LC-5060 recycling HPLC apparatus equipped with two JAIGEL-2HR columns, using $CHCl_3$ (containing EtOH) as eluent.

Instrumental

^{1}H (500 MHz) and ^{13}C (126 MHz) NMR spectra were recorded on a JEOL ECZ500R spectrometer. Chemical shifts were reported as the delta scale in ppm relative to the internal standards (δ = 7.26 ppm for ^{1}H and 77.16 ppm for ^{13}C in $CDCl_3$).

High-resolution (HR) atmospheric pressure chemical ionization (APCI) orbitrap mass spectra and **electrospray ionization (ESI) orbitrap mass spectra** were recorded on a Thermo Fisher Scientific EXACTIVE Plus instrument using the APCI and ESI methods respectively in positive ion modes. **High-resolution electron ionization (HR-EI) selector mass spectra** were recorded on a JEOL JMS-SX102A instrument using the EI method in positive ion mode.

Gel permeation chromatography (GPC) analysis was performed at 25 °C on a JASCO high performance liquid chromatography (HPLC) apparatus composed of a PU-4180 RHPLC pump, DG-4000-04 degassing unit, 7725i manual injector, a CO-4060 column oven, and an UV-4075 UV–vis detector, by equipping two Shodex GPC LF804 columns (Ø = 8.0 mm, *l* = 300 mm). Molecular weight was calculated with a ChromNAV ver. 2 software using a Shodex standard (type: SM-105). Stabilizer-free tetrahydrofuran (THF) was purchased from FUJIFILM Wako Pure Chemical Industry, Ltd.

Powder X-ray diffraction (PXRD) measurements were performed on a Rigaku MiniFlex II diffractometer with Cu-Kα radiation (λ = 1.54187 Å).

Differential scanning calorimetry (DSC) curves were recorded on a Hitachi DSC7020. **Thermogravimetric analysis (TGA)** results were obtained by a Hitachi STA7200.

Gas adsorption measurements were conducted using a MicrotracBEL BELSORP-max-12-N-VP-K apparatus.

All calculations were carried out using the Gaussian 16 program.[S4] The structures were fully optimized without any symmetry restrictions. The calculations were performed by the density functional theory (DFT) method with ωB97X-D level [S5], employing a basis set 6-31G(d,p).

2. Synthetic procedures and compound data

Table S201. Attempted formylation of [3.3.3]- and [4.3.3]propellanes via lithium and magnesium reagents.

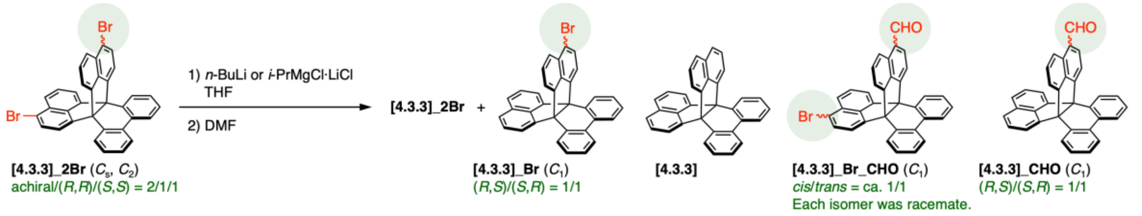

entry	*starting material*	*reagent (equiv)*	*conditions*	results
1	**[4.3.3]_2Br**	1) *n*-BuLi (2.5)<br>2) DMF (>3)	1) THF, −80 °C, 30 min<br>2) to RT	complicated mixture<br>**[4.3.3]_CHO** (trace)
2	**[4.3.3]_2Br**	1) *n*-BuLi (1.05)<br>2) DMF (>3)	1) THF, −80 °C, 50 min<br>2) to RT	complicated mixture<br>**[4.3.3]_Br_CHO** (trace)
3	**[4.3.3]_2Br**	1) *n*-BuLi (2.0)<br>2) DMF (5)	1) Et_2O, −30 °C, 2 h<br>2) to RT, 11 h	complicated mixture<br>trace aldehyde peak in ^{1}H NMR
4	**[4.3.3]_2Br**	1) *i*-PrMgCl·LiCl (3.0)<br>2) DMF (>5)	1) THF, RT, 5 h<br>2) to RT, 24 h	S.M. recovery<br>trace aldehyde peak in ^{1}H NMR
5	**[4.3.3]_2Br**	1) *i*-PrMgCl·LiCl (1.2)<br>2) DMF (>3)	1) THF, RT, 5 h<br>2) to RT, 24 h	S.M. recovery<br>trace aldehyde peak in ^{1}H NMR
6	**[4.3.3]_2Br**	1) *i*-PrMgCl·LiCl (3.0)<br>2) DMF (>5)	1) THF, 40 °C, 3 h<br>2) 40 °C, 12 h	S.M. and de-bromination<br>weak aldehyde peak in ^{1}H NMR
7	**[4.3.3]_2Br**	1) *i*-PrMgCl·LiCl (23)<br>2) DMF (25)	1) THF, 50 °C, 23 h<br>2) 50 °C, 26 h	S.M. and de-bromination<br>**[4.3.3]_CHO** (trace)

Dibromo[4.3.3]propellane **[4.3.3]_2Br** was reacted with 2.5 equivalents of *n*-buthyllithium (*n*-BuLi) in THF at −80 °C to generate the organolithium species, which was quenched with *N*,*N*-dimethylformamide (DMF) (Table S201, entry 1). After the reaction, no target compound **[4.3.3]_2CHO** was observed in the crude ^{1}H NMR spectrum. The mixture was quite complicated and trace monoformyl product **[4.3.3]_CHO** was detected due to debromination. To minimize decomposition caused by excess organolithium species, *n*-BuLi was reduced to 1.05 equivalent (entry 2). However, the change did not improve the situation very much, giving only trace amounts of target **[4.3.3]_Br_CHO**. A reaction in Et_2O did not provide successful formylation either, with a weak aldehyde ^{1}H NMR signal (entry 3). Then, we envisioned using a mild magnesium reagent instead of *n*-BuLi. When we conducted halogen–metal exchange with iPrMgCl·LiCl at room temperature, recovery of the starting material was predominant after quenching with DMF (entries 4, 5). The exchange at higher temperature and increase of the equivalents of iPrMgCl·LiCl did not lead to full

conversion of **[4.3.3]_2Br**, and partial debromination was observed instead of considerable progress of the intended reaction (entries 6, 7).

Table S202. Attempted electrophilic formylation of [3.3.3]- and [4.3.3]propellanes.

entry	*starting material*	*reagent (equiv)*	*conditions*	results
1	**[4.3.3]_Br** (mixture)[a]	DMF (1.5) $POCl_3$ (1.5)	1,2-dichloroethane, 80 °C, 6 h	S.M. recovery
2[b]	**[4.3.3]_Br** (mixture)[a]	HMTA (2.0)	TFA, reflux, 48 h	S.M. recovery (major) mono/di-formylation (trace)[c]
3	**[4.3.3]**	HMTA (3.0)	TFA, reflux, 15 h	complicated mixture mono/di-formylation (trace)[c]

[a] **[4.3.3]** was brominated with 1.05 equiv of Br_2, which was used the reaction in entries 1, 2.

[b] HMTA, hexamethylenetetramine; TFA, trifluoroacetic acid.

[c] Samples were impure even after chromatographic separation on silica gel.

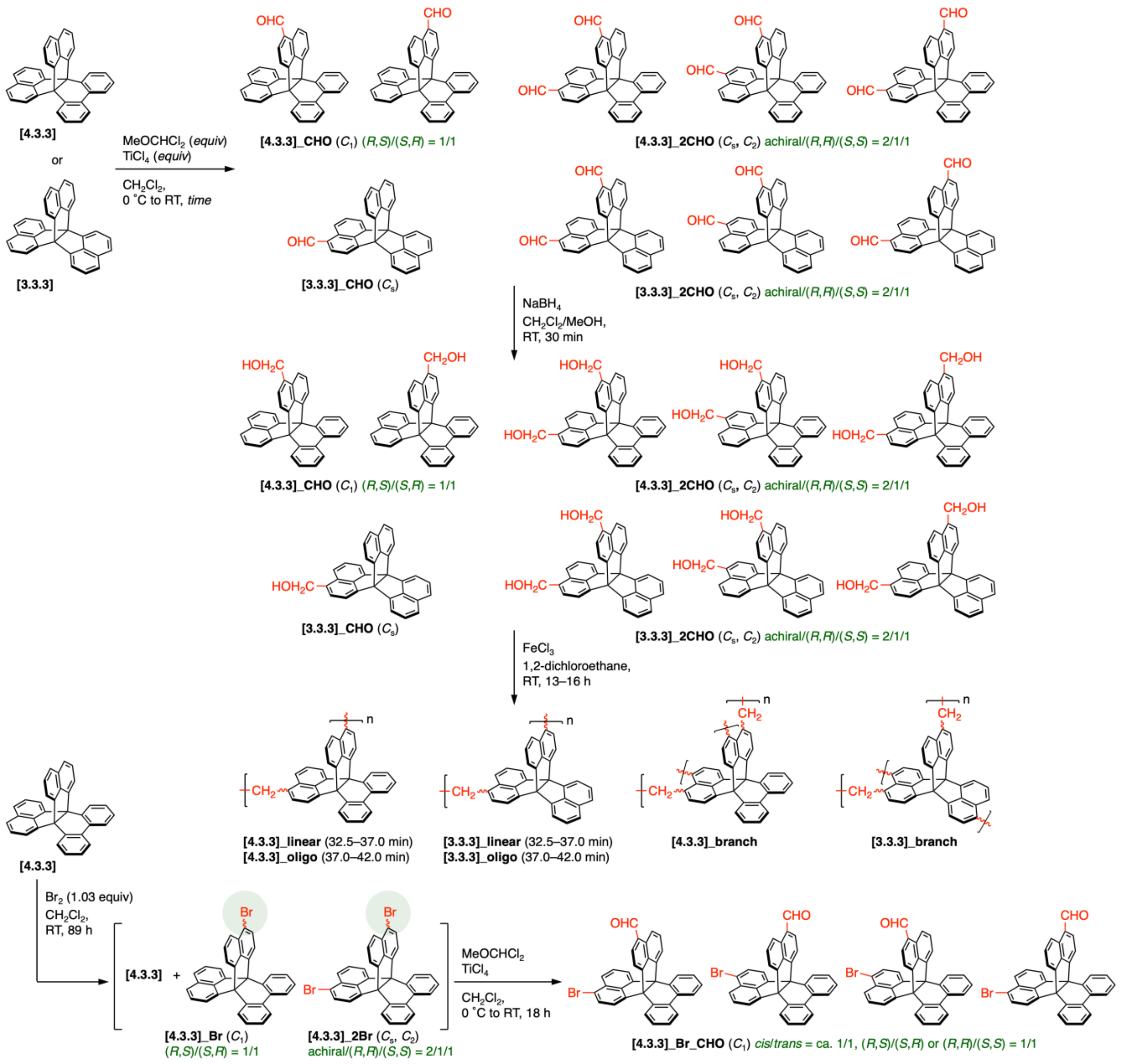

Scheme S201. Formylation[S6] of naphthalene-fused propellanes and their conversion to methylene-alternating copolymers. A possible bonding was displayed for the co-polymers.

Monoformylated [4.3.3]propellane [4.3.3]_CHO: [4.3.3]Propellane **[4.3.3]** (214 mg, 0.50 mmol) was dissolved in CH_2Cl_2 (5.0 mL) in a 100-mL round-bottomed flask under nitrogen atmosphere. After dichloromethyl methyl ether (0.054 mL, 0.60 mmol) was added, the mixture was cooled to 0 °C with an ice bath. Then, $TiCl_4$ (0.066 mL, 0.60 mmol) was added dropwise. The mixture was allowed to warm to room temperature and stirred for 1.5 h. The mixture was poured into a mixture of ice and deionized water and extracted with CH_2Cl_2 three times. The organic extract was washed with $NaHCO_3$ (aq) and dried over anhydrous Na_2SO_4. After the solvent was evaporated under reduced pressure, purification by column chromatography on Wakogel C-400HG (CH_2Cl_2/*n*-hexane 1:1 to 1:0) afforded off-white solids (183 mg, 0.40 mmol, 80%). ^{1}H NMR ($CDCl_3$, 298 K): δ/ppm = 10.24 (s, 1H, C<u>H</u>O) 8.78 (dd, *J* = 8.5, 1.0 Hz, 1H, 7-Naph^{CHO}-H), 8.30 (m, 2H,

3,3'-Biph-H), 7.99–7.95 (m, 3H, 6,6'-Biph-H (2H), 2-Naph^{CHO}-H (1H)), 7.80–7.76 (m, 2H, 3,5-Naph^{CHO}-H), 7.70–7.75 (m, 3H, 6-Naph^{CHO}-H (1H), 2,7-Naph-H (2H)), 7.62–7.59 (m, 2H, 4,5-Naph-H), 7.52–7.43 (m, 4H, 4,4'-Biph-H (2H), 3,6-Naph-H (2H)), 7.37–7.32 (m, 2H, 5,5'-Biph-H); ^{13}C NMR ($CDCl_3$, 298 K): δ/ppm = 192.9, 155.9, 148.6, 147.5, 147.3, 138.8, 136.9, 136.3, 135.6, 135.0, 131.8, 131.6, 131.3, 131.2, 129.6, 128.9, 128.9, 128.8, 128.4, 128.3, 128.2, 128.0, 127.9, 127.8, 124.3, 124.3, 124.2, 124.1, 122.7, 122.4, 121.0, 120.7, 120.1, 67.8, 67.7; HR-APCI-orbitrap-MS (positive): *m*/*z* calcd for $[C_{35}H_{21}O_1]^+$: 457.1587 $[M+H]^+$; found: 457.1586.

Monoformylated [3.3.3]propellane [3.3.3]_CHO: [3.3.3]Propellane **[3.3.3]** (201 mg, 0.50 mmol) was dissolved in CH_2Cl_2 (5.0 mL) in a 100-mL round-bottomed flask under nitrogen atmosphere. After dichloromethyl methyl ether (0.054 mL, 0.60 mmol) was added, the mixture was cooled to 0 °C with an ice bath. Then, $TiCl_4$ (0.066 mL, 0.60 mmol) was added dropwise. The mixture was allowed to warm to room temperature and stirred for 1.5 h. The mixture was poured into a mixture of ice and deionized water and extracted with CH_2Cl_2 three times. The organic extract was washed with $NaHCO_3$ (aq) and dried over anhydrous Na_2SO_4. After the solvent was evaporated under reduced pressure, purification by column chromatography on Wakogel C-400HG (CH_2Cl_2/*n*-hexane 1:1 to 1:0) afforded off-white solids (145 mg, 0.34 mmol, 67%). ^{1}H NMR ($CDCl_3$, 298 K): δ/ppm = 10.25 (s, 1H, C$\underline{H}$O), 8.82 (dd, *J* = 8.5, 0.5 Hz, 1H, 7-Naph^{CHO}-H), 8.18–8.15 (m, 2H, 2,5-Naph^{CHO}-H), 8.06–8.02 (m, 5H, 3-Naph^{CHO}-H (1H), 2,7-Naph-H (4H)), 7.75 (dd, *J* = 8.5, 7.0 Hz, 1H, 6-Naph^{CHO}-H), 7.66–7.62 (m, 4H, 4,5-Naph-H), 7.58–7.54 (m, 4H, 3,6-Naph-H); ^{13}C NMR ($CDCl_3$, 298 K): δ/ppm = 192.9, 154.4, 147.0, 146.1, 145.5, 139.4, 137.6, 137.2, 132.6, 131.9, 129.9, 129.7, 128.8, 128.6, 124.8, 124.6, 123.1, 120.9, 119.4, 118.7, 79.9, 79.6; HR-APCI-orbitrap-MS (positive): *m*/*z* calcd for $[C_{33}H_{19}O_1]^+$: 431.1430 $[M+H]^+$; found: 431.1432.

Diformylated [3.3.3]propellane [3.3.3]_2CHO: [3.3.3]Propellane **[3.3.3]** (201 mg, 0.50 mmol) was dissolved in CH_2Cl_2 (5.0 mL) in a 100-mL round-bottomed flask under nitrogen atmosphere. After dichloromethyl methyl ether (0.11 mL, 1.2 mmol) was added, the mixture was cooled to 0 °C with an ice bath. Then, $TiCl_4$ (0.13 mL, 1.2 mmol) was added dropwise. The mixture was allowed to warm to room temperature and stirred for 1.5 h. The mixture was poured into a mixture of ice and deionized water and extracted with CH_2Cl_2 three times. The organic extract was washed with $NaHCO_3$ (aq) and dried over anhydrous Na_2SO_4. After the solvent was evaporated under reduced pressure, purification by column chromatography on Wakogel C-400HG (CH_2Cl_2/*n*-hexane 2:1 to 1:0, then 10% acetone was added) afforded off-white solids (58 mg, 0.13 mmol, 25%) along with **[3.3.3]_CHO** (132 mg, 0.31 mmol, 61%). ^{1}H NMR ($CDCl_3$, 298 K): δ/ppm = 10.26–10.24 (m, 2H, C$\underline{H}$O), 8.88–8.82 (m, 2H, 7-Naph^{CHO}-H), 8.19–8.16 (m, 4H, 2,5-Naph^{CHO}-H), 8.07–8.02 (m, 4H, 3-Naph^{CHO}-H (2H), 2,7-Naph-H (2H)), 7.78–7.74 (m, 2H, 6-Naph^{CHO}-H), 7.70 (m, 2H, 4,5-Naph-H), 7.61–7.57 (m, 2H, 3,6-Naph-H); ^{13}C NMR ($CDCl_3$, 298 K): δ/ppm = 192.8, 192.7, 153.6, 153.0, 146.3, 145.7, 145.4, 144.8, 144.3, 139.5, 139.2, 137.5, 137.0, 132.6, 132.0, 131.9, 130.3, 130.1, 129.7, 128.9, 128.8, 128.7, 125.3, 125.1, 124.9, 123.7,

123.4, 121.0, 121.0, 119.6, 118.9, 79.8, 79.4, 79.1; HR-APCI-orbitrap-MS (positive): *m*/*z* calcd for $[C_{34}H_{19}O_2]^+$: 459.1380 $[M+H]^+$; found: 459.1380.

Diformylated [4.3.3]propellane [4.3.3]_2CHO: [4.3.3]Propellane **[4.3.3]** (214 mg, 0.50 mmol) was dissolved in CH_2Cl_2 (5.0 mL) in a 100-mL round-bottomed flask under nitrogen atmosphere. After dichloromethyl methyl ether (0.11 mL, 1.2 mmol) was added, the mixture was cooled to 0 °C with an ice bath. Then, $TiCl_4$ (0.14 mL, 1.2 mmol) was added dropwise. The mixture was allowed to warm to room temperature and stirred for 18 h. The mixture was poured into a mixture of ice and deionized water and extracted with CH_2Cl_2 three times. The organic extract was washed with $NaHCO_3$ (aq) and dried over anhydrous Na_2SO_4. After the solvent was evaporated under reduced pressure, purification by column chromatography on Wakogel C-400HG (CH_2Cl_2/*n*-hexane 2:1 to 1:0, then 10% acetone was added) afforded off-white solids (24 mg, 0.050 mmol, 9.9%) along with **[4.3.3]_CHO** (76 mg, 0.17 mmol, 33%). Purification of **[4.3.3]_2CHO** was not completely successful despite repeated attempts with chromatography on silica gel, GPC–HPLC, and precipitation from a mixture of CH_2Cl_2 and MeOH. We speculate that the side product may have a formyl group on the biphenyl segment instead of naphthalene one. ^1H NMR ($CDCl_3$, 298 K): δ/ppm = 10.26–10.25 (m, 2H, C<u>H</u>O), 8.83–8.79 (m, 2H, 7-Naph^{CHO}-H), 8.28–8.24 (m, 2H, 3,3'-Biph-H), 8.03–7.96 (m, 4H, 6,6'-Biph-H (2H), 2-Naph^{CHO}-H (2H)), 7.81–7.76 (m, 4H, 3,5-Naph^{CHO}-H), 7.73–7.67 (m, 2H, 6-Naph^{CHO}-H), 7.49–7.45 (m, 2H, 4,4'-Biph-H) 7.39–7.36 (m, 2H, 5,5'-Biph-H); ^{13}C NMR ($CDCl_3$, 298 K): δ/ppm = 192.8, 192.8, 155.0, 154.7, 147.8, 147.6, 138.9, 138.6, 136.8, 136.7, 135.0, 134.4, 134.9, 131.8, 131.5, 131.4, 131.8, 131.1, 130.0, 129.9, 129.0, 129.0, 128.8, 128.8, 128.7, 128.6, 128.5, 128.5, 128.3, 128.2, 128.1, 124.6, 124.5, 124.4, 123.2, 123.0, 122.6, 122.4, 120.4, 120.1, 67.9, 67.8, 67.7; HR-APCI-orbitrap-MS (positive): *m*/*z* calcd for $[C_{36}H_{21}O_2]^+$: 485.1536 $[M+H]^+$; found: 485.1535.

Monoformylated bromo[4.3.3]propellane [4.3.3]_Br_CHO: [4.3.3]Propellane **[4.3.3]** (1.29 g, 3.0 mmol) was dissolved in CH_2Cl_2 (75 mL) in a 200-mL round-bottomed flask under nitrogen atmosphere. To the mixture, Br_2 (0.16 mL, 3.1 mmol) was added dropwise. The mixture was stirred at room temperature for 89 h and then quenched with $Na_2S_2O_3$ (aq). The mixture was extracted with CH_2Cl_2 three times. The organic extract was passed through a short column of anhydrous Na_2SO_4 and Wakosil 60. Evaporation of the solvent under reduced pressure gave a mixture of starting material **[4.3.3]**, monobromo[4.3.3]propellane **[4.3.3]_Br**, and dibrominated products **[4.3.3]_2Br** (1.18 g, ca. 2.32 mmol, ca. 77%). The crude brominated [4.3.3]propellane (507 mg, ca. 1.0 mmol) was dissolved in CH_2Cl_2 (10 mL) in a 100-mL round-bottomed flask under nitrogen atmosphere. After dichloromethyl methyl ether (0.11 mL, 1.2 mmol) was added, the mixture was cooled to 0 °C with an ice bath. Then, $TiCl_4$ (0.13 mL, 1.2 mmol) was added dropwise. The mixture was allowed to warm to room temperature and stirred for 18 h. The mixture was poured into a mixture of ice and deionized water and extracted with CH_2Cl_2 three times. The organic extract was washed with $NaHCO_3$ (aq) and dried

over anhydrous Na_2SO_4. After the solvent was evaporated under reduced pressure, purification by column chromatography on Wakogel C-400HG (CH_2Cl_2/*n*-hexane 1:1 to 1:0) afforded off-white solids (268 mg, 0.50 mmol, 39% in 2 steps). Purification of **[4.3.3]_Br_CHO** was not completely successful despite repeated attempts with chromatography on silica gel, GPC-HPLC, and precipitation from a mixture of CH_2Cl_2 and MeOH. We speculate that the side product may have a formyl group on the biphenyl segment instead of naphthalene one. ^{1}H NMR ($CDCl_3$, 298 K): δ/ppm = 10.21 and 10.20 (s, 1H, C<u>H</u>O) 8.81–8.77 (m, 1H, 7-Naph^{CHO}-H), 8.32–8.22 (m, 2H, 3,3'-Biph-H), 7.97–7.89 (m, 3H, 6,6'-Biph-H (2H), 2-Naph^{CHO}-H (1H)), 7.81–7.64 (m, 6H, 3,5,6-Naph^{CHO}-H (3H), 2,5,7-Naph^{Br}-H (2H)), 7.61–7.56 (m, 1H, 6-Naph^{Br}-H), 7.52–7.44 (m, 3H, 4,4'-Biph-H (2H), 3-Naph^{Br}-H (2H)), 7.37–7.32 (m, 2H, 5,5'-Biph-H); ^{13}C NMR ($CDCl_3$, 298 K): δ/ppm = 192.8, 192.8, 155.8, 155.2, 155.1, 148.5, 148.0, 147.9, 147.7, 147.5, 147.5, 147.4, 147.3, 147.1, 138.8, 138.7, 138.7, 137.1, 136.9, 136.8, 136.8, 136.3, 135.5, 135.1, 135.0, 134.9, 134.5, 134.4, 131.8, 131.6, 131.5, 131.3, 131.2, 131.2, 131.2, 131.2, 131.1, 131.1, 131.1, 129.7, 129.7, 129.6, 129.5, 129.3, 128.9, 128.9, 128.8, 128.8, 128.8, 128.7, 128.6, 128.5, 128.4, 128.4, 128.3, 128.2, 128.1, 128.1, 128.0, 127.9, 127.9, 127.9, 127.7, 124.4, 124.4, 124.3, 124.3, 124.3, 124.2, 124.1, 123.8, 123.7, 122.9, 122.6, 122.4, 122.3, 122.0, 121.8, 121.7, 121.6, 121.0, 120.7, 120.1, 120.0, 119.2, 118.9, 67.9, 67.8, 67.8, 67.7, 67.3, 67.2; HR-APCI-orbitrap-MS (positive): *m*/*z* calcd for $[C_{35}H_{20}{}^{79}Br_1O_1]^+$: 535.0692 $[M+H]^+$; found: 535.0697.

Monohydroxymethylated [3.3.3]propellane [3.3.3]_CH₂OH: Monoformylated [3.3.3]propellane **[3.3.3]_CHO** (215 mg, 0.50 mmol) was dissolved in a mixture of CH_2Cl_2 (20 mL) and MeOH (20 mL) in a 200-mL round-bottomed flask. After sodium borohydride ($NaBH_4$, 25 mg, 0.65 mmol) was added, the mixture was stirred at room temperature for 30 min. The mixture was quenched with deionized water, NH_4Cl (aq), and then dilute HCl (aq). The mixture was extracted with CH_2Cl_2 three times. The organic extract was dried over anhydrous Na_2SO_4. After the solvent was evaporated under reduced pressure, purification by column chromatography on Wakogel C-400HG (CH_2Cl_2/*n*-hexane 2:1 to 1.0) afforded off-white solids (217 mg, 0.50 mmol, 100%). ^{1}H NMR ($CDCl_3$, 298 K): δ/ppm = 8.07 (d, *J* = 8.5 Hz, 1H, 7-Naph^{CH2OH}-H), 8.04–8.01 (m, 4H, 2,7-Naph-H), 8.00 (d, *J* = 9.0 Hz, 1H, 2-Naph^{CH2OH}-H), 7.84 (d, *J* = 8.5 Hz, 1H, 5-Naph^{CH2OH}-H), 7.62–7.59 (m, 5H, 6-Naph^{CH2OH}-H (1H), 4,5-Naph-H (4H)), 7.57–7.52 (m, 5H, 3-Naph^{CH2OH}-H (1H), 3,6-Naph-H (4H)), 5.01 (s, 2H, C<u>H₂</u>OH), 1.54 (s, 1H, CH₂O<u>H</u>); ^{13}C NMR ($CDCl_3$, 298 K): δ/ppm = 148.7, 148.6, 148.2, 148.1, 136.9, 136.5, 136.0, 136.0, 133.8, 131.8, 131.4, 131.4, 129.9, 128.9, 128.9, 128.4, 128.2, 128.2, 128.0, 127.5, 127.3, 124.1, 123.8, 120.9, 120.8, 120.7, 120.6, 120.4, 67.8, 67.3, 63.1; HR-ESI-orbitrap-MS (positive): *m*/*z* calcd for $[C_{33}H_{20}O_1Na_1]^+$: 455.1406 $[M+Na]^+$; found: 455.1398.

Monohydroxymethylated [4.3.3]propellane [4.3.3]_CH₂OH: Monoformylated [4.3.3]propellane **[4.3.3]_CHO** (228 mg, 0.50 mmol) was dissolved in a mixture of CH_2Cl_2 (20 mL) and MeOH (20 mL) in a 200-mL round-bottomed flask. After sodium borohydride ($NaBH_4$, 25 mg, 0.65 mmol) was added, the mixture was

stirred at room temperature for 30 min. The mixture was quenched with deionized water, NH_4Cl (aq), and then dilute HCl (aq). The mixture was extracted with CH_2Cl_2 three times. The organic extract was dried over anhydrous Na_2SO_4. After the solvent was evaporated under reduced pressure, purification by column chromatography on Wakogel C-400HG (CH_2Cl_2/*n*-hexane = 2/1 to 1/0) afforded off-white solids (213 mg, 0.46 mmol, 93%). ^1H NMR ($CDCl_3$, 298 K): δ/ppm = 8.34–8.31 (m, 2H, 3,3'-Biph-H), 7.95 (d, *J* = 8.5 Hz, 2H, 6,6'-Biph-H), 7.77 (d, *J* = 8.5 Hz, 1H, 7-Naph^{CH2OH}-H), 7.69 (d, *J* = 7.0 Hz, 1H, 5-Naph^{CH2OH}-H), 7.65 (d, 7.0 Hz, 2H, 2,7-Naph-H), 7.60 (d, *J* = 7.0 Hz, 1H, 2-Naph^{CH2OH}-H), 7.58–7.56 (m, 2H, 4,5-Naph-H), 7.51 (dd, *J* = 8.5, 7.0 Hz, 1H, 6-Naph^{CH2OH}-H), 7.49–7.42 (m, 5H, 4,4'-Biph-H (2H), 3-Naph^{CH2OH}-H (1H), 3,6-Naph-H (2H)), 7.32 (t, *J* = 8.5 Hz, 2H, 5,5'-Biph-H), 4.96 (d, *J* = 6.0 Hz, 2H, C$\underline{H_2}$OH), 1.58 (t, *J* = 6.0 Hz, 1H, $CH_2O\underline{H}$); ^{13}C NMR ($CDCl_3$, 298 K): δ/ppm = 147.3, 147.2, 146.6, 146.6, 137.6, 137.3, 134.4, 132.6, 130.6, 129.0, 128.6, 127.9, 124.4, 121.3, 119.6, 119.2, 119.2, 118.9, 80.1, 79.5, 63.2; HR-ESI-orbitrap-MS (positive): *m*/*z* calcd for $[C_{35}H_{22}O_1Na_1]^+$: 481.1563 $[M+Na]^+$; found: 481.1557.

Dihydroxymethylated [3.3.3]propellane [3.3.3]_2CH$_2$OH: Di-formylated [4.3.3]propellane **[4.3.3]_2CHO** (23 mg, 0.050 mmol) was dissolved in a mixture of CH_2Cl_2 (5.0 mL) and MeOH (5.0 mL) in a 100-mL round-bottomed flask. After sodium borohydride ($NaBH_4$, ca. 5 mg, ca. 0.13 mmol) was added, the mixture was stirred at room temperature for 30 min. The mixture was quenched with deionized water, NH_4Cl (aq), and then dilute HCl (aq). The mixture was extracted with CH_2Cl_2 three times. The organic extract was dried over anhydrous Na_2SO_4. After the solvent was evaporated under reduced pressure, purification by column chromatography on Wakogel C-400HG (CH_2Cl_2/*n*-hexane 2:1 to 1:0, then 10% acetone was added) afforded off-white solids (16 mg, 0.035 mmol, 69%). ^1H NMR ($CDCl_3$, 298 K): δ/ppm = 8.08–8.06 (m, 2H, 7-Naph^{CH2OH}-H), 8.04–7.98 (m, 4H, 2-Naph^{CH2OH}-H (2H), 2,7-Naph-H (2H)), 7.83 (d, *J* = 8.5 Hz, 2H, 5-Naph^{CH2OH}-H), 7.62–7.58 (m, 4H, 6-Naph^{CH2OH}-H (2H), 4,5-Naph-H (2H)), 7.56–7.52 (m, 4H, 3-Naph^{CH2OH}-H (2H), 3,6-Naph-H (2H)), 5.01 (s, 4H, C$\underline{H_2}$OH); ^{13}C NMR ($CDCl_3$, 298 K): δ/ppm = 147.1, 147.1, 147.0, 146.5, 146.4, 146.4, 137.6, 137.2, 134.5, 132.6, 130.6, 129.0, 128.6, 127.9, 124.4, 121.3, 119.6, 119.2, 118.9, 79.5, 78.9, 63.2; HR-ESI-orbitrap-MS (positive): *m*/*z* calcd for $[C_{34}H_{22}O_2Na_1]^+$: 485.1512 $[M+Na]^+$; found: 485.1505.

Dihydroxymethylated [4.3.3]propellane [4.3.3]_2CH$_2$OH: [4.3.3]Propellane **[4.3.3]** (2.01 g, 4.70 mmol) was dissolved in CH_2Cl_2 (47 mL) in a 200-mL round-bottom flask under nitrogen atmosphere. After dichloromethyl methyl ether (1.02 mL, 11.3 mmol) was added, the mixture was cooled to 0 °C with an ice bath. Then, $TiCl_4$ (1.24 mL, 11.3 mmol) was added dropwise. The mixture was allowed to warm to room temperature and stirred for 1 h. The mixture was poured into a mixture of ice and deionized water and extracted with CH_2Cl_2 three times. The organic extract was washed with $NaHCO_3$ (aq) and dried over anhydrous Na_2SO_4. After the solvent was evaporated under reduced pressure, purification by column chromatography on Wakogel C-400HG (CH_2Cl_2/*n*-hexane 1:2 to 1:0) afforded a mixture of **[4.3.3]_2CHO** and

[4.3.3]_CHO (424 mg) as off-white solids along with pure **[4.3.3]_CHO** (1.68 g, 3.68 mmol, 78%). The mixture was dissolved in a mixture of CH_2Cl_2 (11 mL) and MeOH (11 mL) in a 200-mL round-bottomed flask. After sodium borohydride ($NaBH_4$, ca. 80 mg) was added in portions, the mixture was stirred at room temperature for 30 min. The mixture was quenched with deionized water, NH_4Cl (aq), and then dilute HCl (aq). The mixture was extracted with CH_2Cl_2 three times. The organic extract was dried over anhydrous Na_2SO_4. After the solvent was evaporated under reduced pressure, purification by column chromatography on Wakogel C-400HG (CH_2Cl_2/*n*-hexane 2:1 to 1:0, then 10–50% acetone was added) afforded off-white solids (140 mg, 0.287 mmol, 6.1% in 2 steps). Purification of **[4.3.3]_2CH$_2$OH** was not completely successful despite repeated attempts with chromatography on silica gel, GPC-HPLC, and precipitation from a mixture of CH_2Cl_2 and MeOH. ^{1}H NMR ($CDCl_3$, 298 K): δ/ppm = 8.31–8.28 (m, 2H, 3,3'-Biph-H), 7.96–7.93 (m, 2H, 6,6'-Biph-H), 7.80–7.77 (m, 2H, 7-Naph^{CH2OH}-H), 7.68 (d, *J* = 7.0 Hz, 2H, 5-Naph^{CH2OH}-H), 7.60 (d, *J* = 7.0 Hz, 2H, 2-Naph^{CH2OH}-H), 7.55–7.51 (m, 2H, 6-Naph^{CH2OH}-H), 7.48 (d, *J* = 7.0 Hz, 2H, 3-Naph^{CH2OH}-H), 7.45–7.41 (m, 2H, 4,4'-Biph-H), 7.34–7.30 (m, 2H, 5,5'-Biph-H), 5.01 (s, 4H, C$\underline{H_2}$OH), 1.57 (s, 2H, CH$_2$O$\underline{H}$); ^{13}C NMR ($CDCl_3$, 298 K): δ/ppm = 148.6, 148.5, 148.5, 136.8, 135.8, 133.9, 131.4, 129.9, 128.9, 128.4, 128,2, 127.6, 127.3, 124.2, 121.0, 120.9, 120.9, 120.5, 120.4, 67.4, 66.9, 63.2; HR-ESI-orbitrap-MS (positive): *m*/*z* calcd for $[C_{36}H_{24}O_2Na_1]^+$: 511.1669 $[M+Na]^+$; found: 511.1666.

General procedure for acid-mediated polymerization of monohydroxymethylated propellanes: Monohydroxymethylated propellane (0.50 mmol) was dissolved in dry 1,2-dichloroethane (25 mL) in a 100-mL round-bottomed flask under nitrogen atmosphere. To the stirred solution, anhydrous iron(III) chloride ($FeCl_3$, 8 mg, 0.05 mmol) was added. The mixture was stirred at room temperature for 13 h and quenched by adding MeOH (3.0 mL). After evaporation of the solvent, the residue was passed through a short column of Wakosil 60 using CH_2Cl_2 as eluent. GPC–HPLC separation in the 2nd cycle gave fractions of polymers (32.5–37.0 min) and oligomers (37.0–42.0 min) as off-white solids.

General procedure for acid-mediated polymerization of dihydroxymethylated propellanes: Dihydroxymethylated propellane (0.50 mmol) was dissolved in dry 1,2-dichloroethane (50 mL) in a 100-mL round-bottomed flask under nitrogen atmosphere. To the stirred solution, anhydrous iron(III) chloride ($FeCl_3$, 16 mg, 0.10 mmol) was added. The mixture was stirred at room temperature for 13 h and quenched by adding MeOH (3.0 mL). Solid material was collected by filtration and washed with CH_2Cl_2 (3 mL × 2), H_2O (3 mL × 2), and acetone (3 mL × 5). (For **[4.3.3]_2CH$_2$OH**, the reaction scale was reduced and used 0.12 mmol of **[4.3.3]_2CH$_2$OH**, 0.02 mmol of $FeCl_3$, and 12 mL of 1,2-dichloroethane, due to the limited amount of starting material.)

Table S203. Acid-mediated polymerization of [3.3.3]- and [4.3.3]propellane alcohols.[a]

entry	*starting material*	*reagent (equiv)*	*conditions*	results
1	**[4.3.3]_2CH2OH** (0.050 mmol)	$FeCl_3$ (0.1)	TCE, RT, 13 h	insoluble in $CDCl_3$ > network polymers
2	**[4.3.3]_CH2OH** (0.17 mmol, 10 mM)	$FeCl_3$ (0.1)	TCE, RT, 13 h	^{1}H NMR was consistent but broad. > soluble oligomers/polymers
3	**[4.3.3]_CH2OH** (chiral, fraction 2) (0.055 mmol, 10 mM)	$FeCl_3$ (0.1)	TCE, RT, 16 h	^{1}H NMR was consistent but broad. > soluble oligomers/polymers
4	**[4.3.3]_CH2OH** (0.30 mmol)	$FeCl_3$ (0.10)	DCE, RT, 16 h	polymers, 44 mg oligomers, 64 mg
5	**[3.3.3]_CH2OH** (0.30 mmol)	$FeCl_3$ (0.10)	DCE, RT, 16 h	polymers, 36 mg oligomers, 22 mg
6	**[4.3.3]_CH2OH** (0.50 mmol)	$FeCl_3$ (0.10)	DCE, RT, 13 h	polymers, 22 mg oligomers, 149 mg
7	**[3.3.3]_CH2OH** (0.50 mmol)	$FeCl_3$ (0.10)	DCE, RT, 13 h	polymers, 53 mg oligomers, 49 mg
8	**[4.3.3]_2CH2OH** (0.12 mmol)	$FeCl_3$ (0.2)	DCE, RT, 16 h	network polymers, 40 mg
9	**[3.3.3]_2CH2OH** (0.50 mmol)	$FeCl_3$ (0.20)	DCE, RT, 13 h	network polymers, 107 mg

[a] TCE, 1,1,2,2-tetrachloroethane; DCE, 1,2-dichloroethane.

3. ^{1}H and ^{13}C NMR spectra

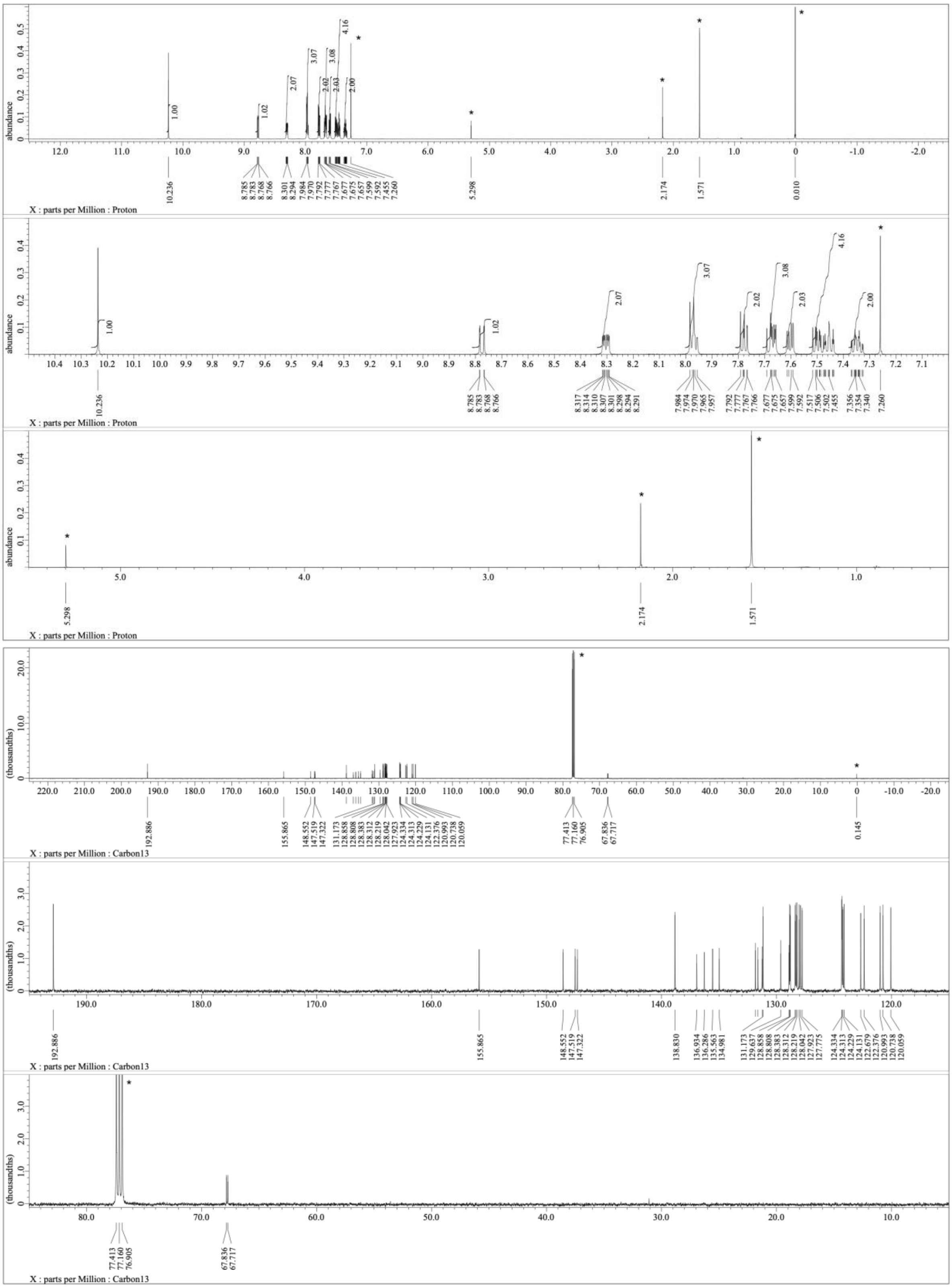

Figure S301. ^{1}H and ^{13}C NMR spectra of **[4.3.3]_CHO** in $CDCl_3$ at room temperature. Peaks marked with * are due to residual solvents and impurities.

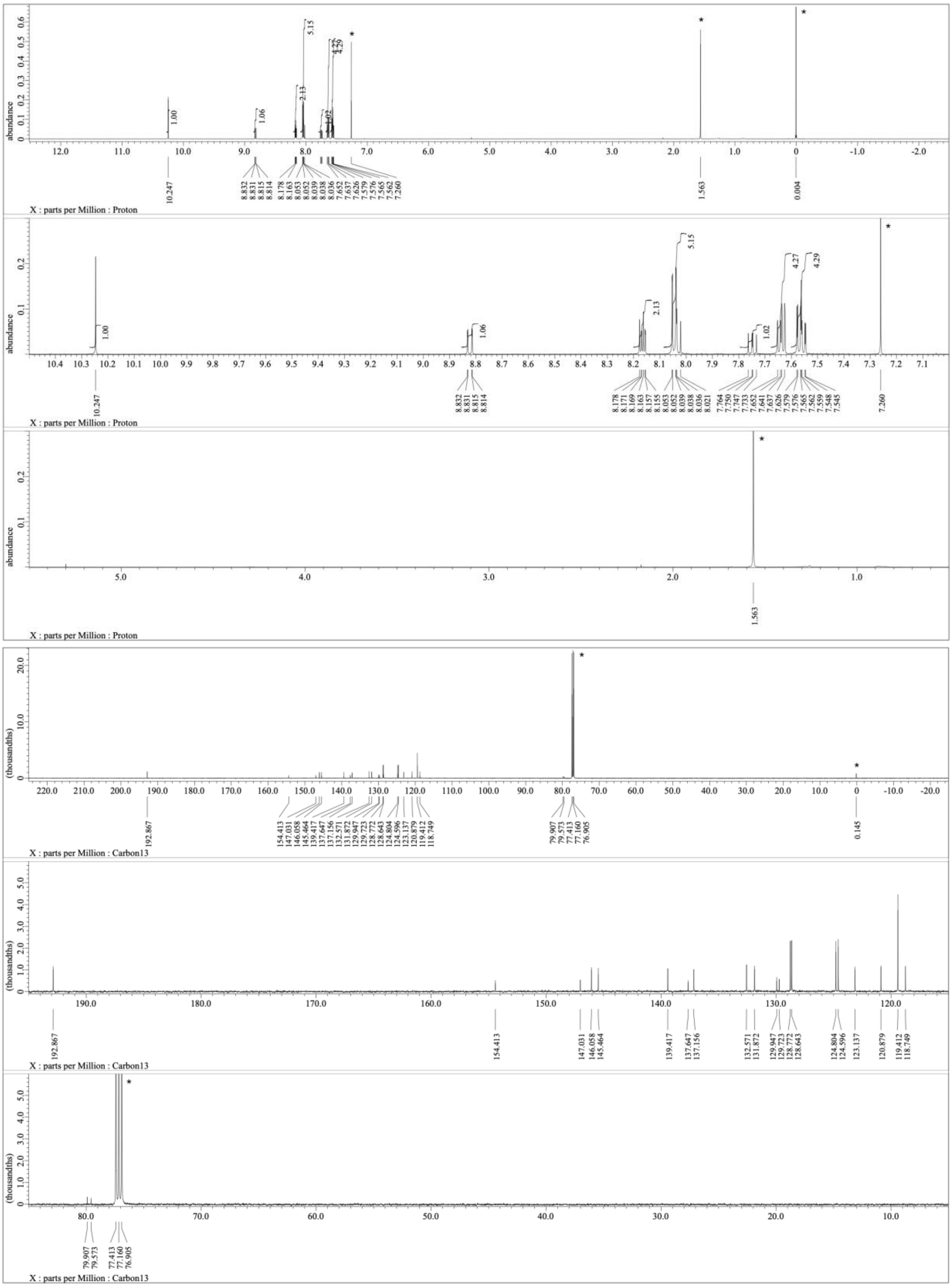

Figure S302. ^{1}H and ^{13}C NMR spectra of **[3.3.3]_CHO** in $CDCl_3$ at room temperature. Peaks marked with * are due to residual solvents and impurities.

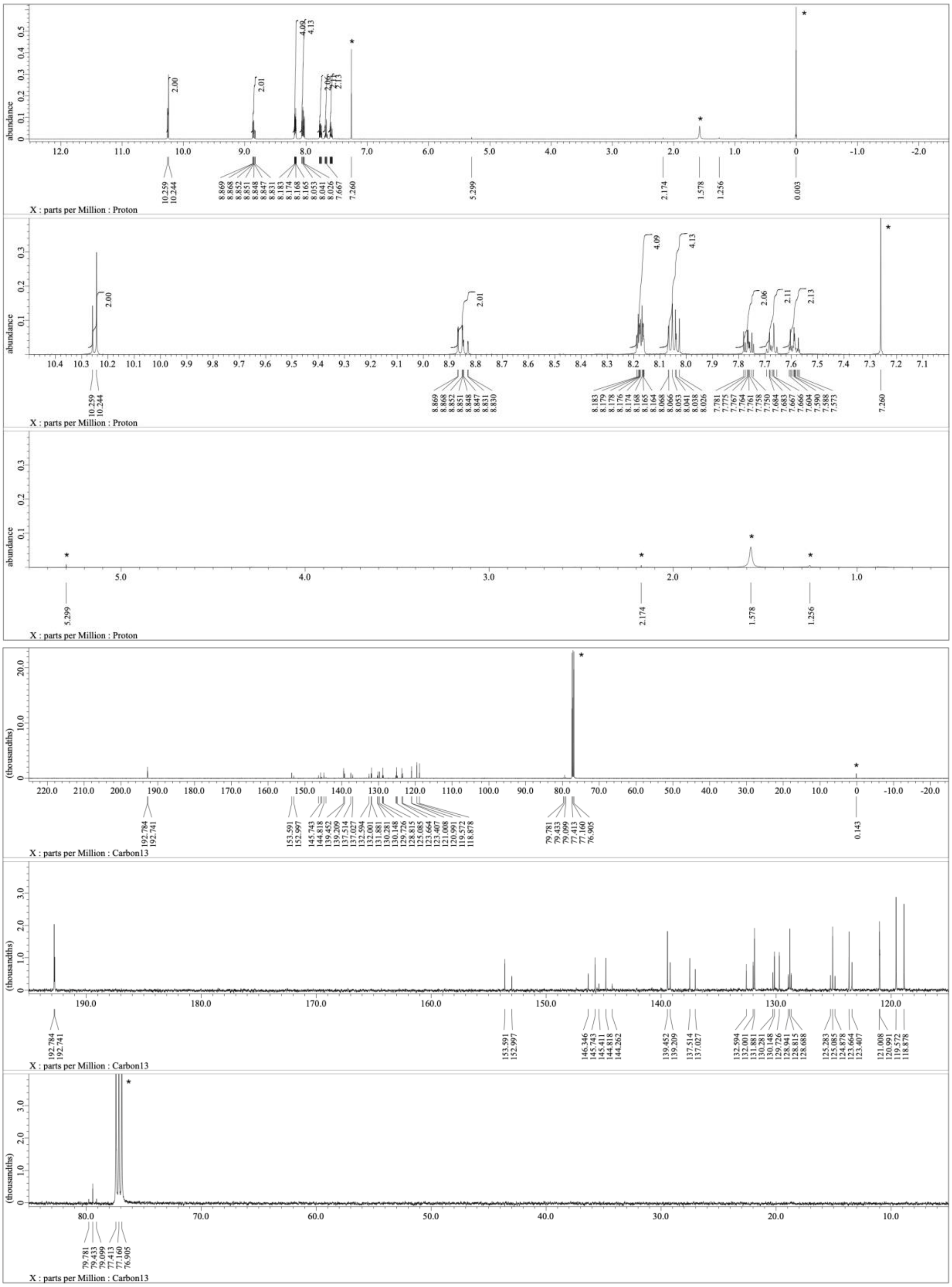

Figure S303. ^{1}H and ^{13}C NMR spectra of **[3.3.3]_2CHO** in $CDCl_3$ at room temperature. Peaks marked with * are due to residual solvents and impurities.

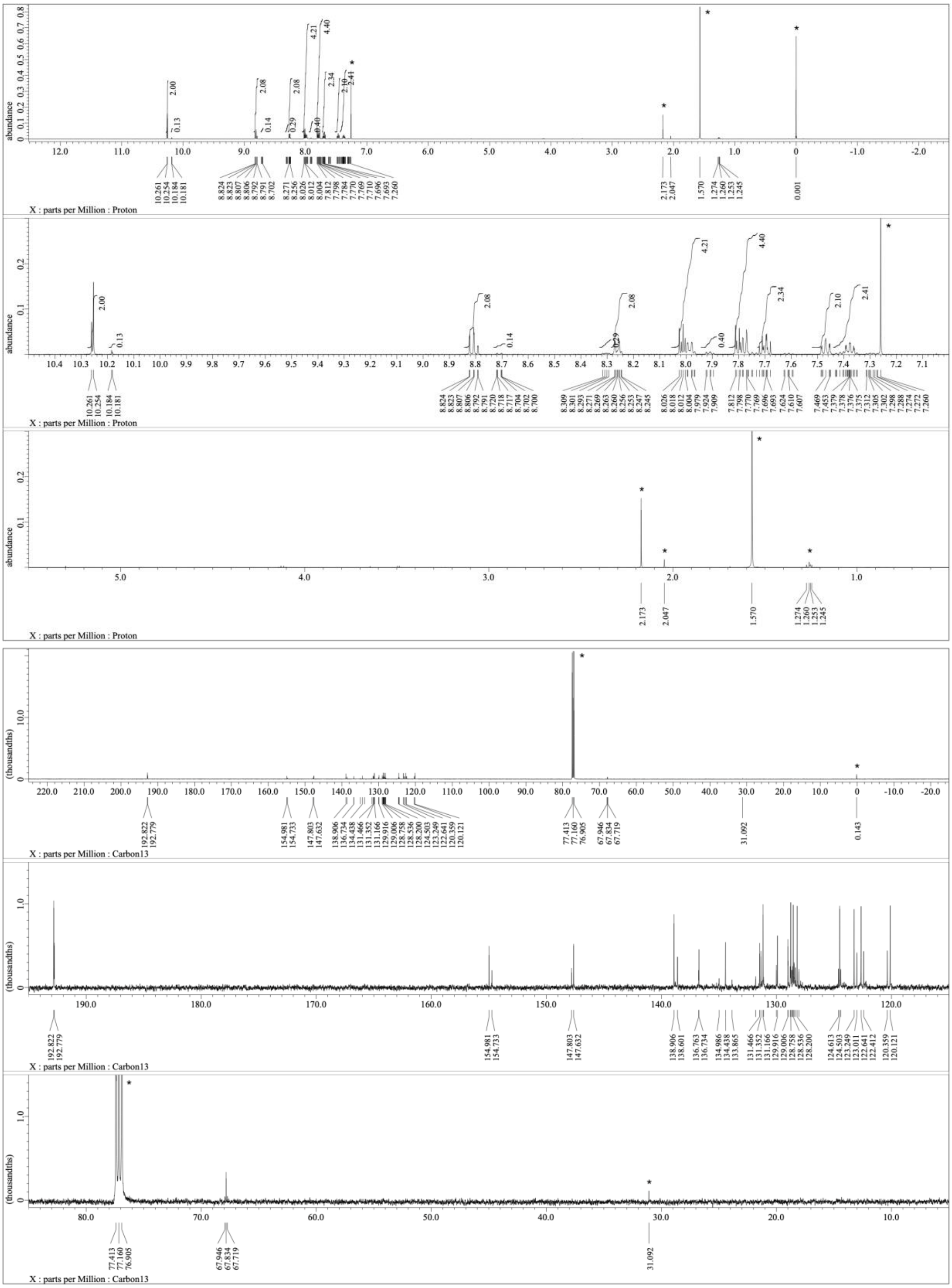

Figure S304. ^{1}H and ^{13}C NMR spectra of **[4.3.3]_2CHO** in $CDCl_3$ at room temperature. Peaks marked with * are due to residual solvents and impurities.

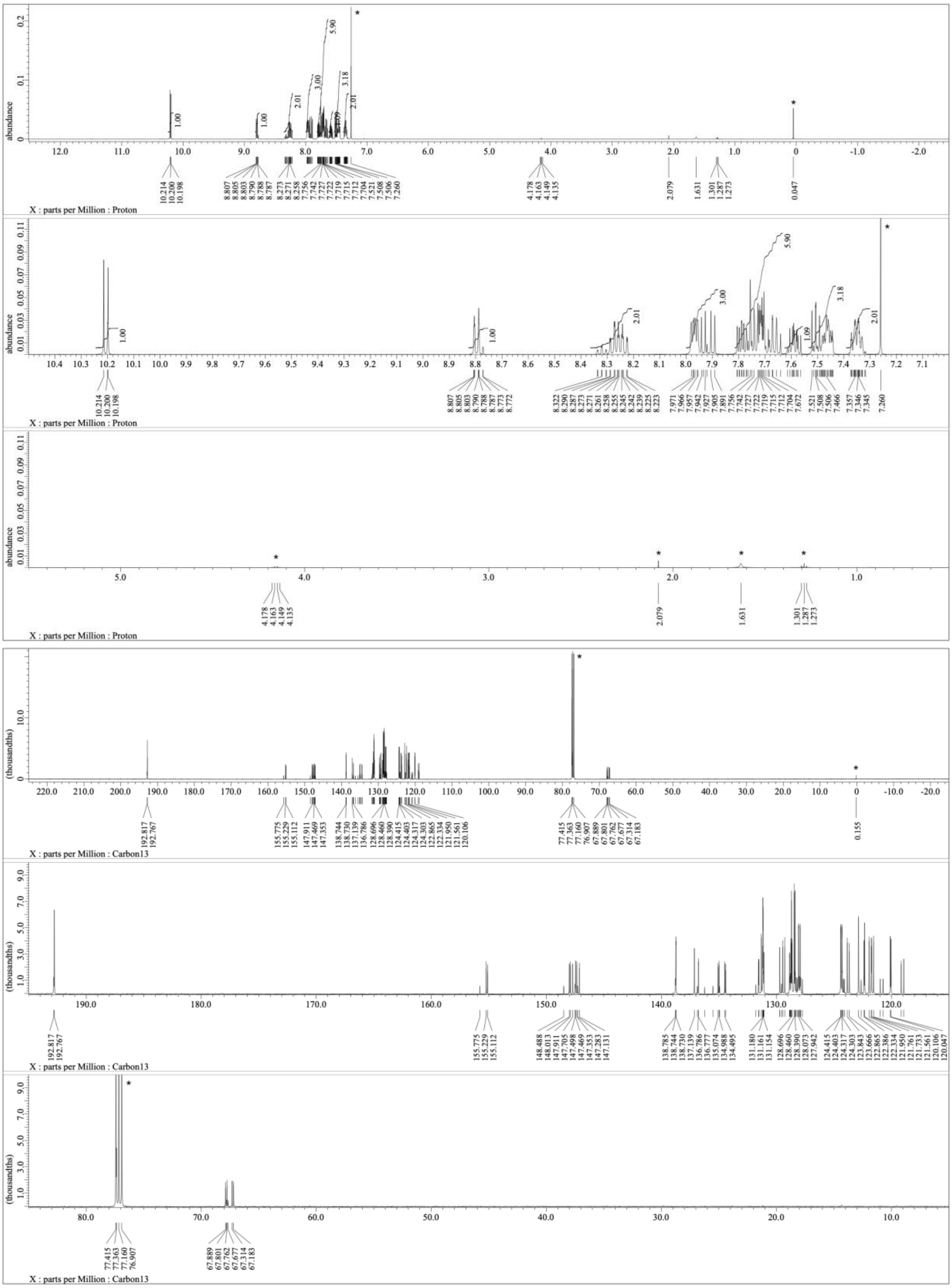

Figure S305. ^{1}H and ^{13}C NMR spectra of **[4.3.3]_Br_CHO** in $CDCl_3$ at room temperature. Peaks marked with * are due to residual solvents and impurities.

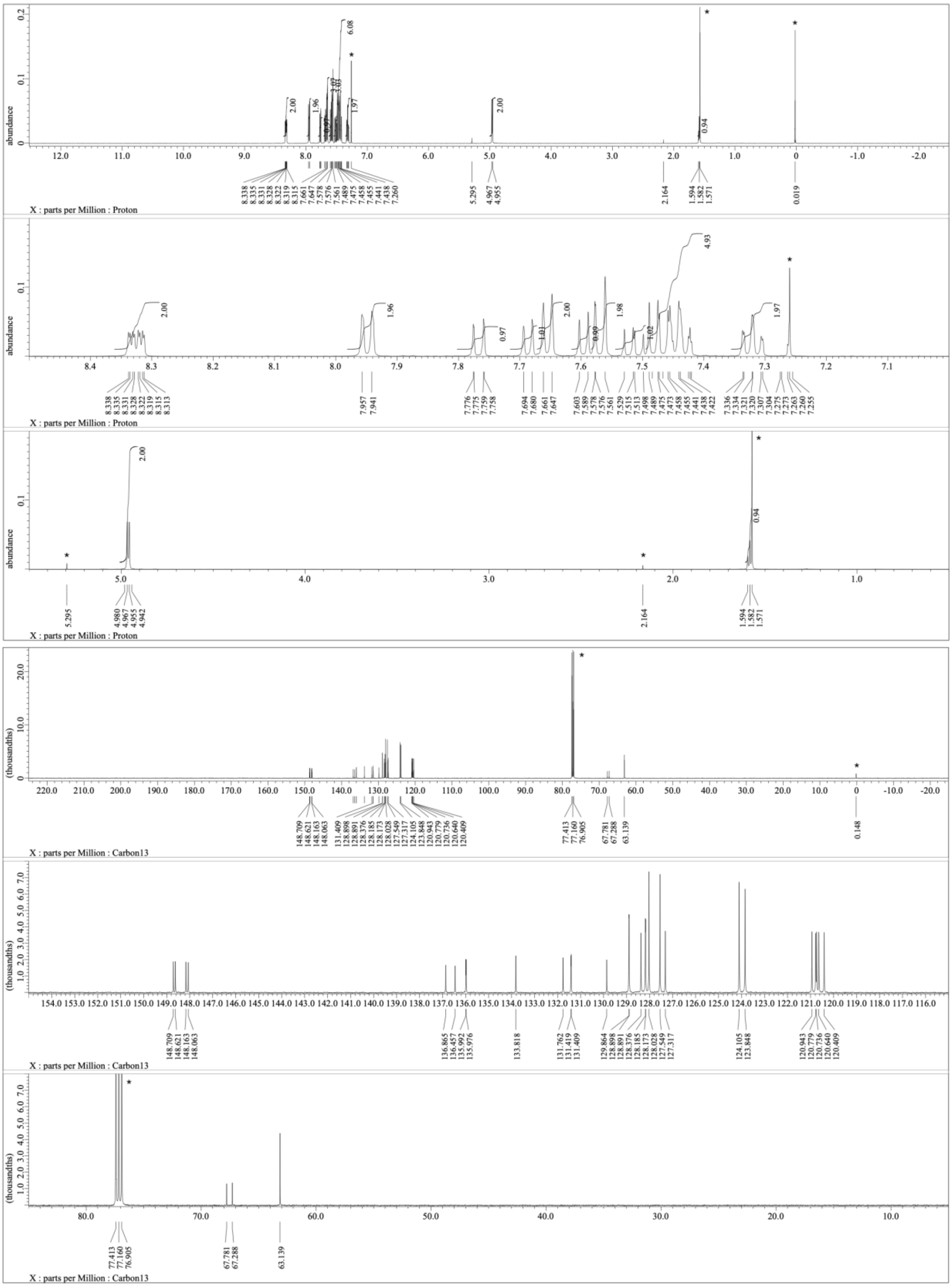

Figure S306. ^{1}H and ^{13}C NMR spectra of **[4.3.3]_CH_2OH** in $CDCl_3$ at room temperature. Peaks marked with * are due to residual solvents and impurities.

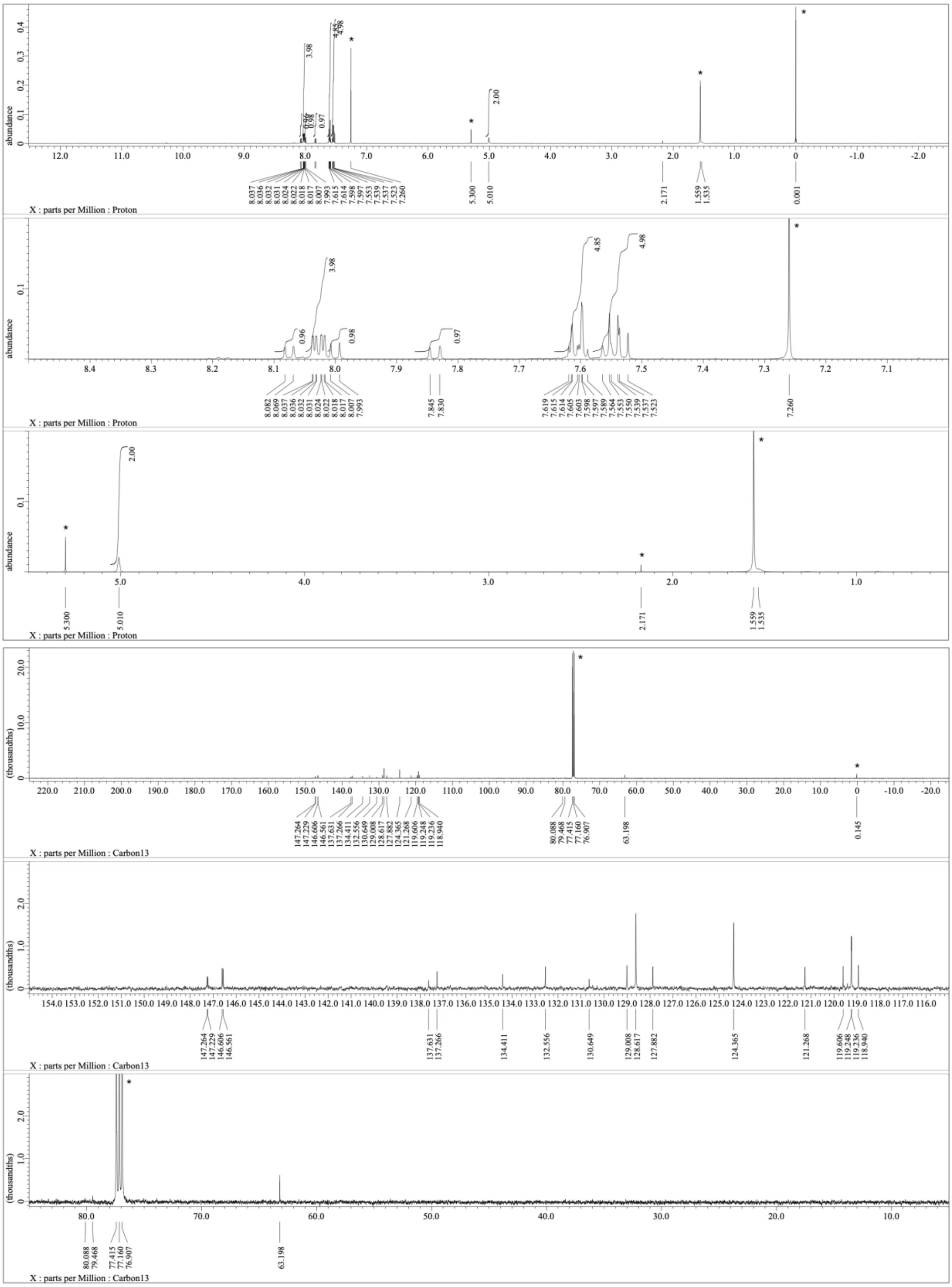

Figure S307. ^{1}H and ^{13}C NMR spectra of **[3.3.3]_CH$_2$OH** in $CDCl_3$ at room temperature. Peaks marked with * are due to residual solvents and impurities.

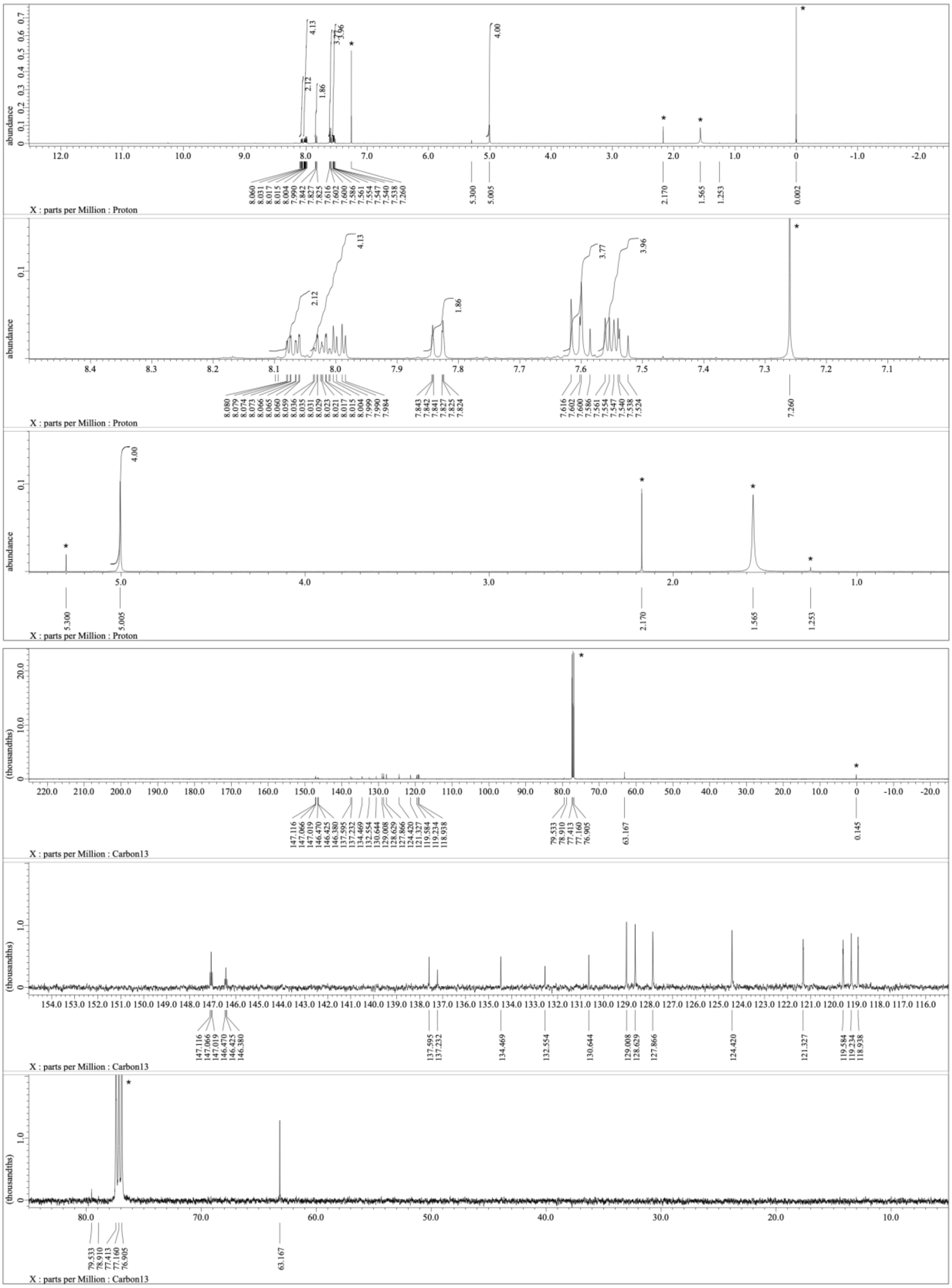

Figure S308. ^{1}H and ^{13}C NMR spectra of **[3.3.3]_2CH₂OH** in $CDCl_3$ at room temperature. Peaks marked with * are due to residual solvents and impurities.

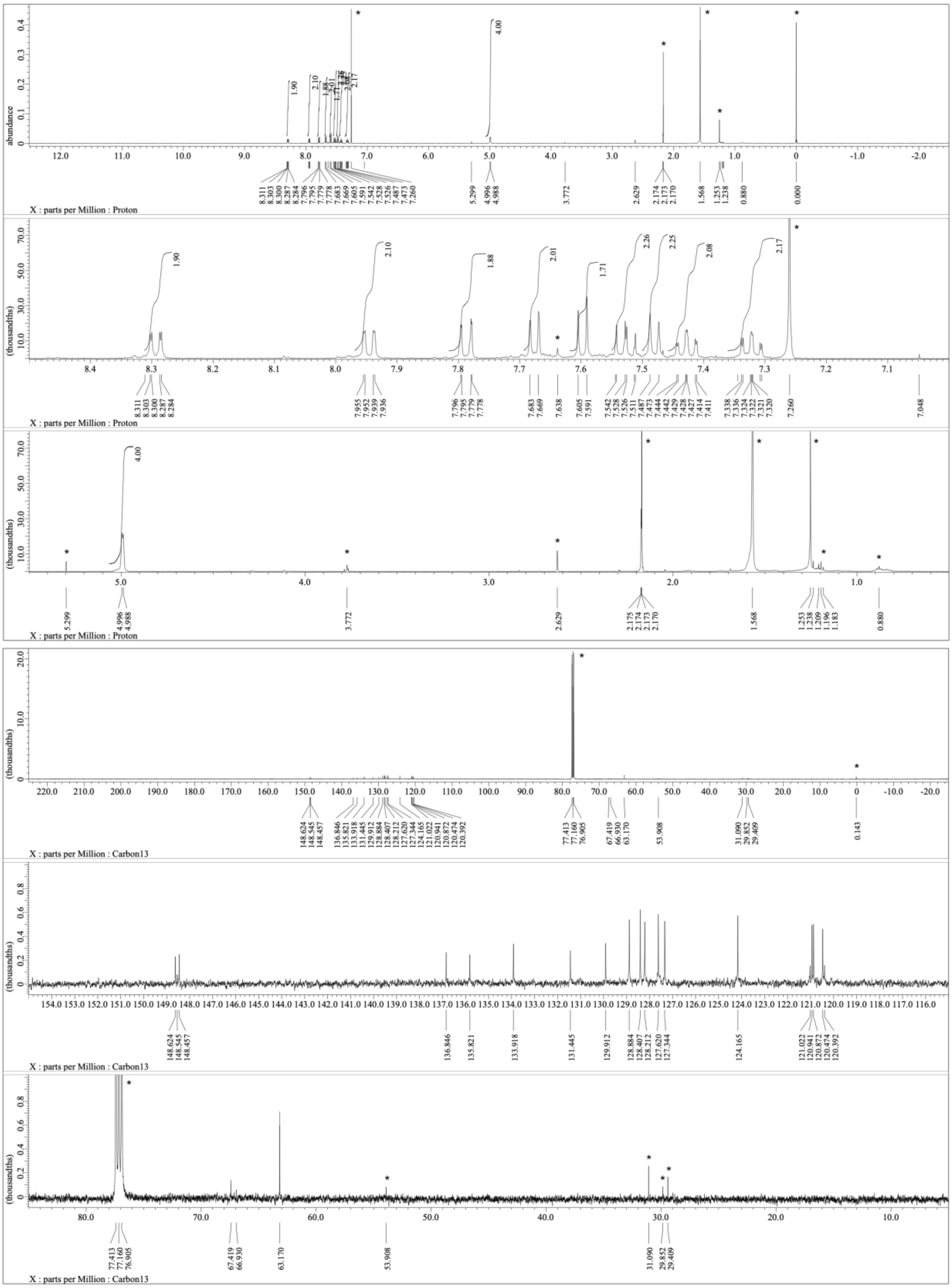

Figure S309. ^{1}H and ^{13}C NMR spectra of **[4.3.3]_2CH₂OH** in $CDCl_3$ at room temperature. Peaks marked with * are due to residual solvents and impurities.

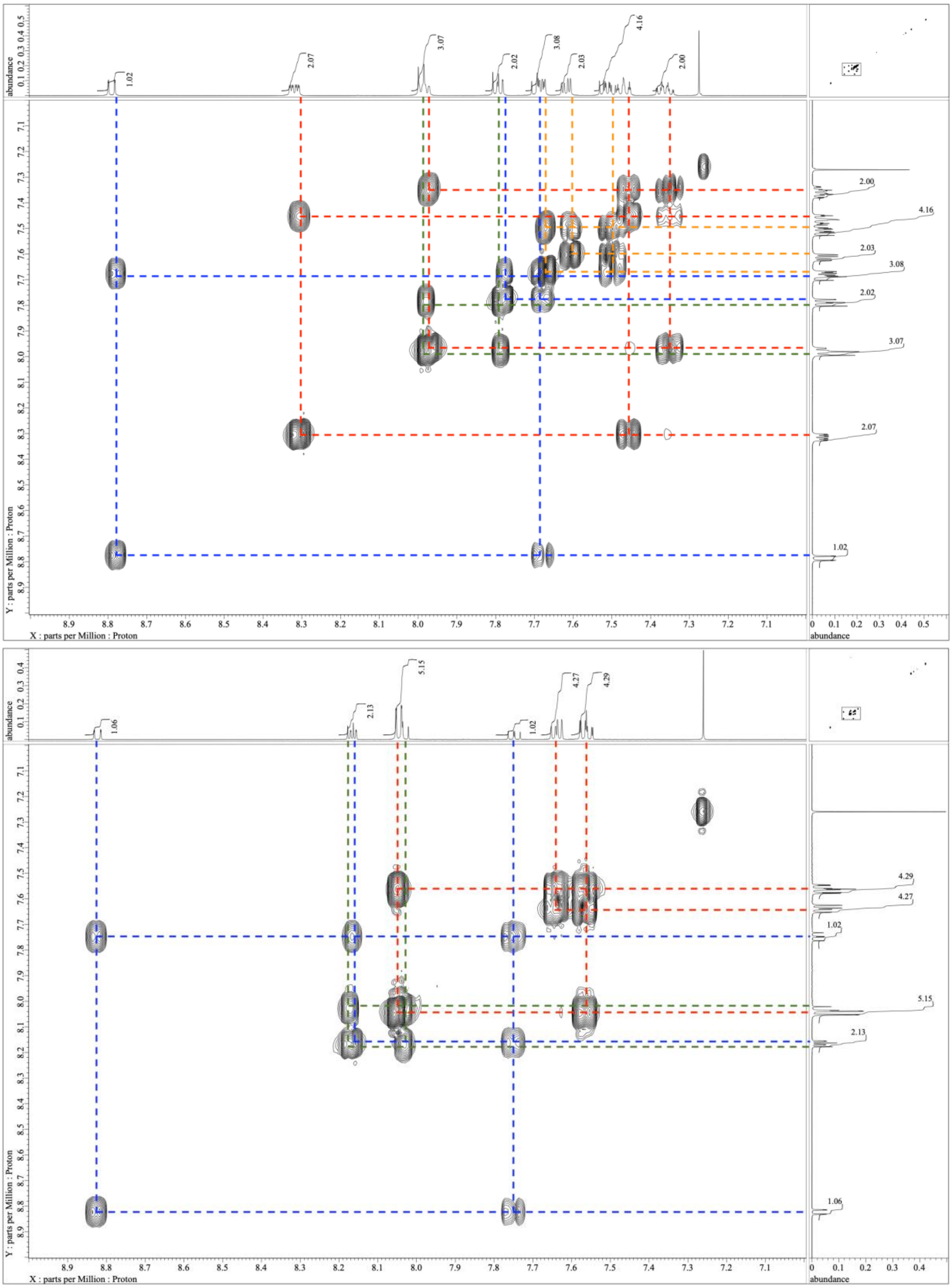

Figure S310. ^{1}H–^{1}H COSY spectra of **[4.3.3]_CHO** (top) and **[3.3.3]_CHO** (bottom) in $CDCl_3$ at room temperature. Peaks marked with * are due to residual solvents and impurities.

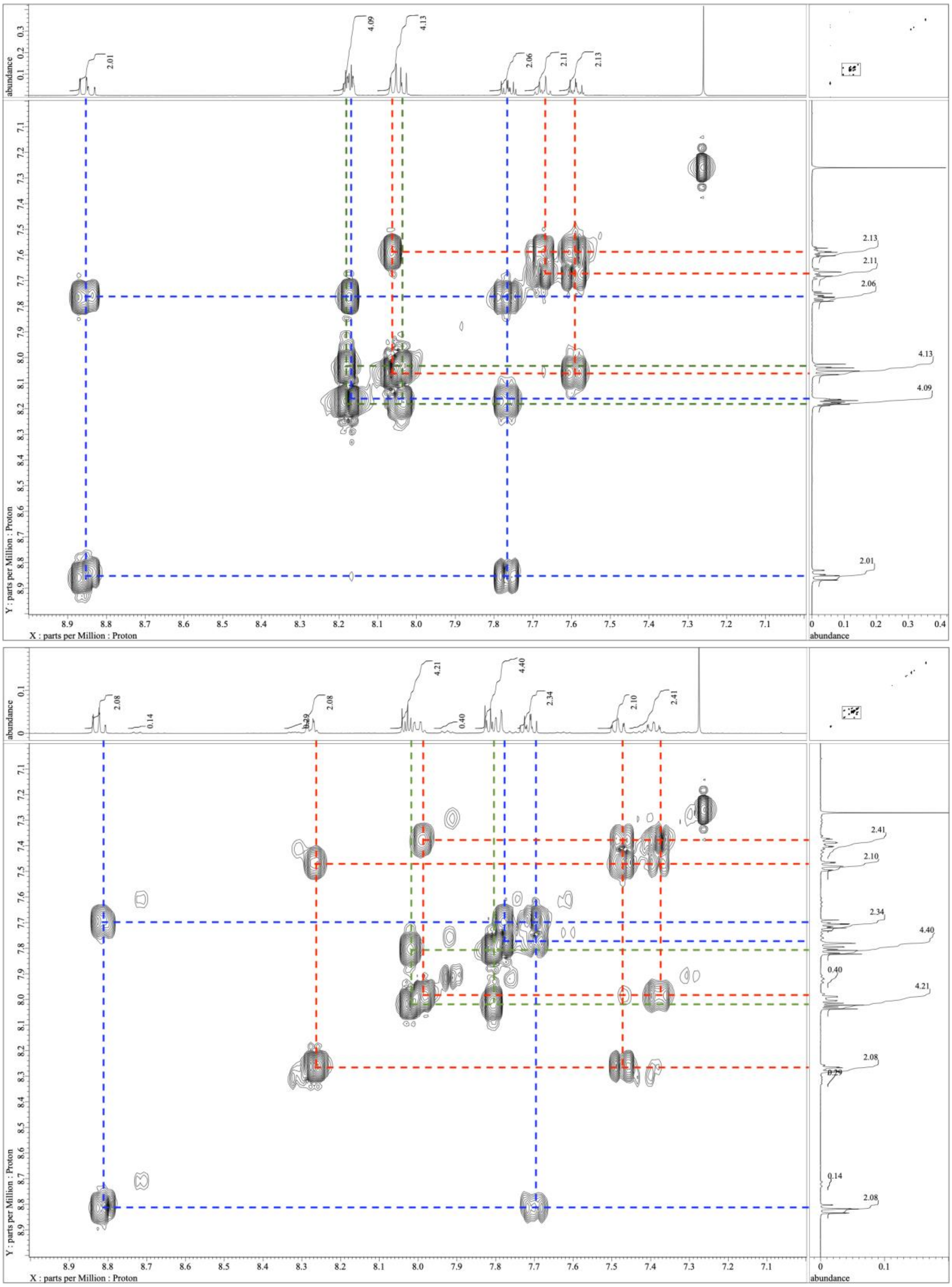

Figure S311. ^{1}H–^{1}H COSY spectra of **[3.3.3]_2CHO** (top) and **[4.3.3]_2CHO** (bottom) in $CDCl_3$ at room temperature. Peaks marked with * are due to residual solvents and impurities.

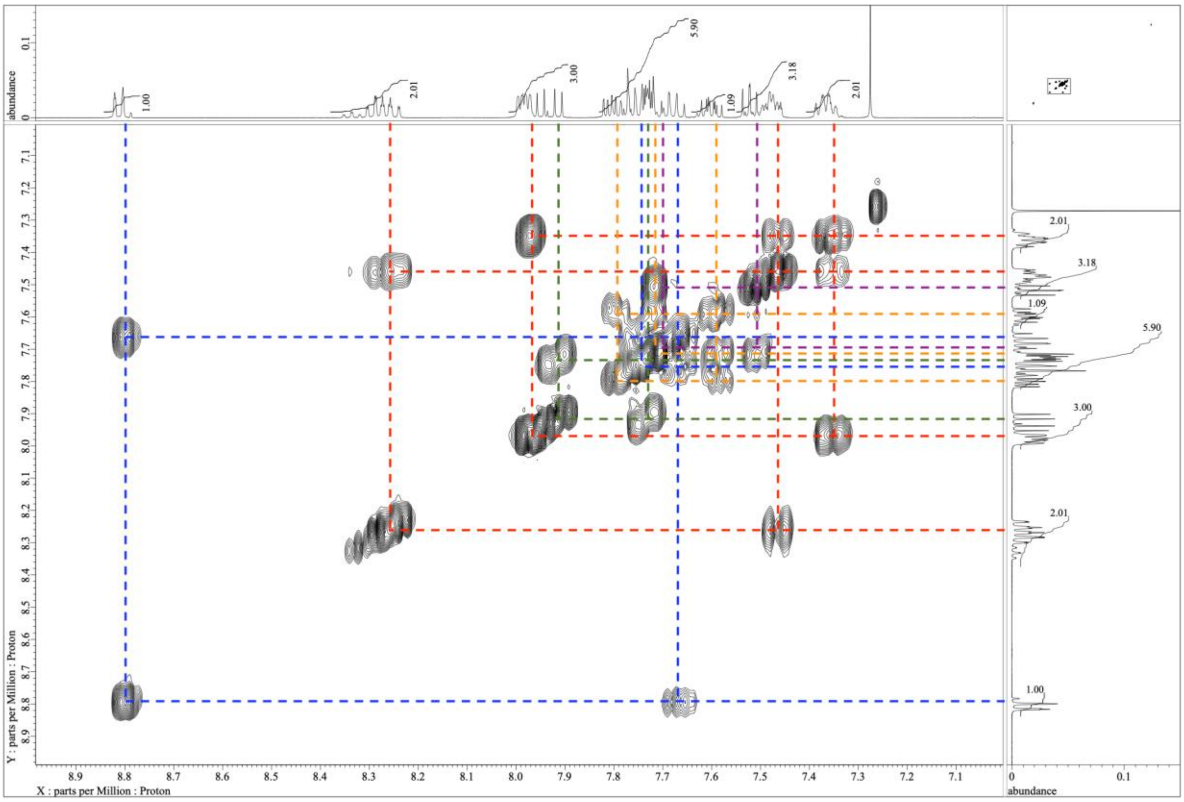

Figure S312. ^{1}H–^{1}H COSY spectra of **[4.3.3]_Br_CHO** in $CDCl_3$ at room temperature. Peaks marked with * are due to residual solvents and impurities.

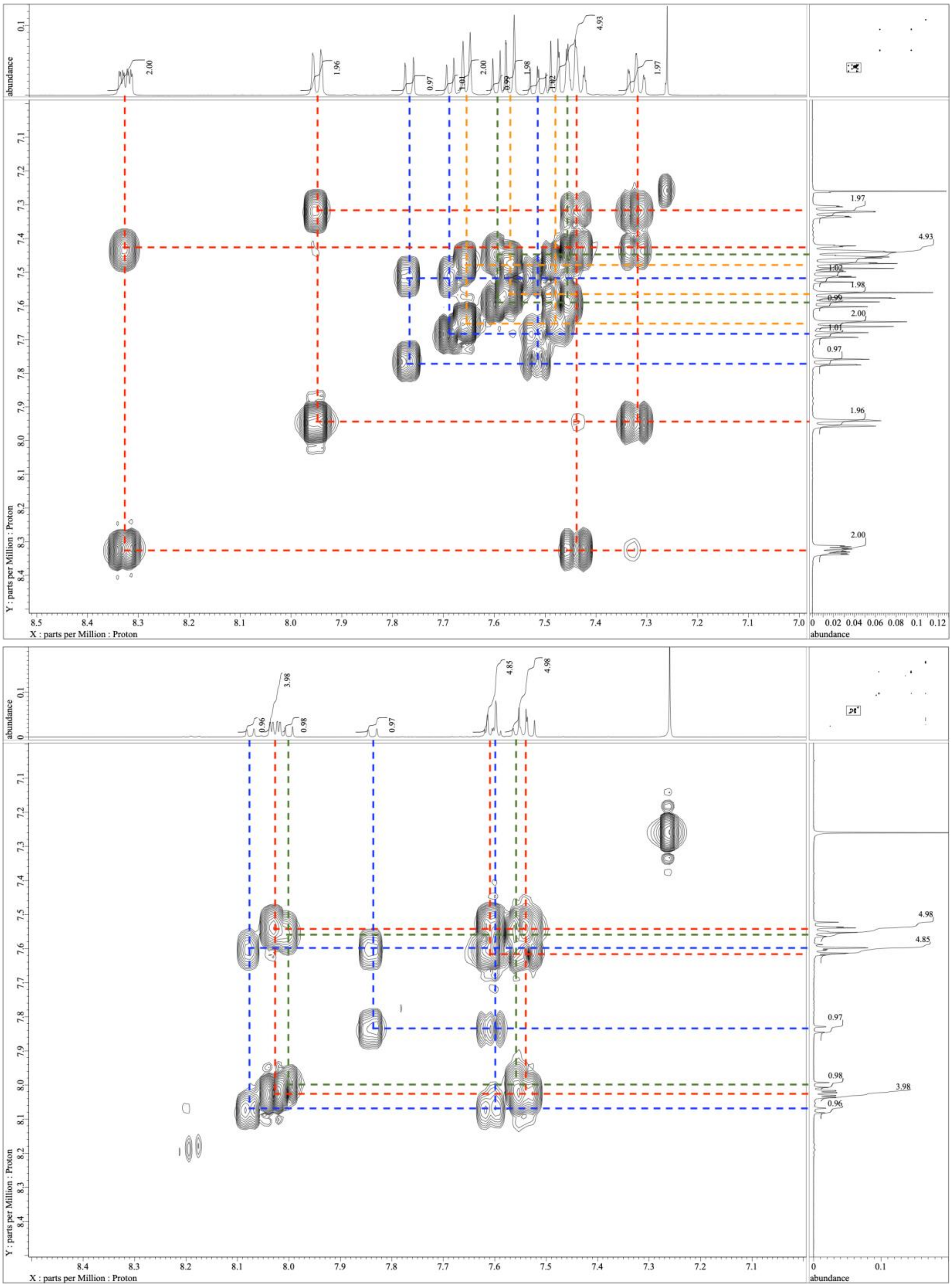

Figure S313. ^1H–^1H COSY spectra of **[4.3.3]_CH$_2$OH** (top) and **[3.3.3]_CH$_2$OH** (bottom) in $CDCl_3$ at room temperature. Peaks marked with * are due to residual solvents and impurities.

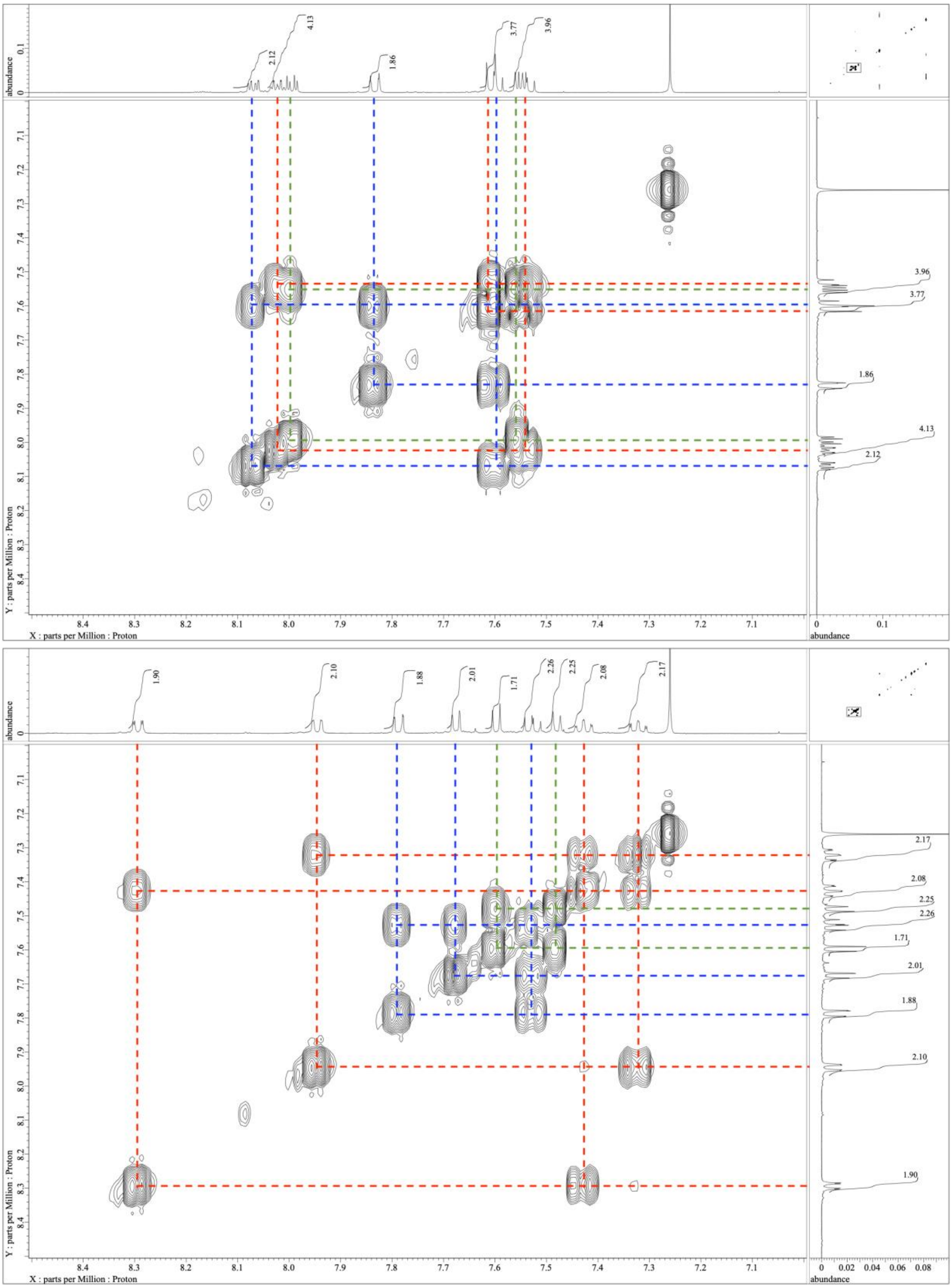

Figure S314. ^1H–^1H COSY spectra of **[3.3.3]_2CH$_2$OH** (top) and **[4.3.3]_2CH$_2$OH** (bottom) in $CDCl_3$ at room temperature. Peaks marked with * are due to residual solvents and impurities.

a)

b)

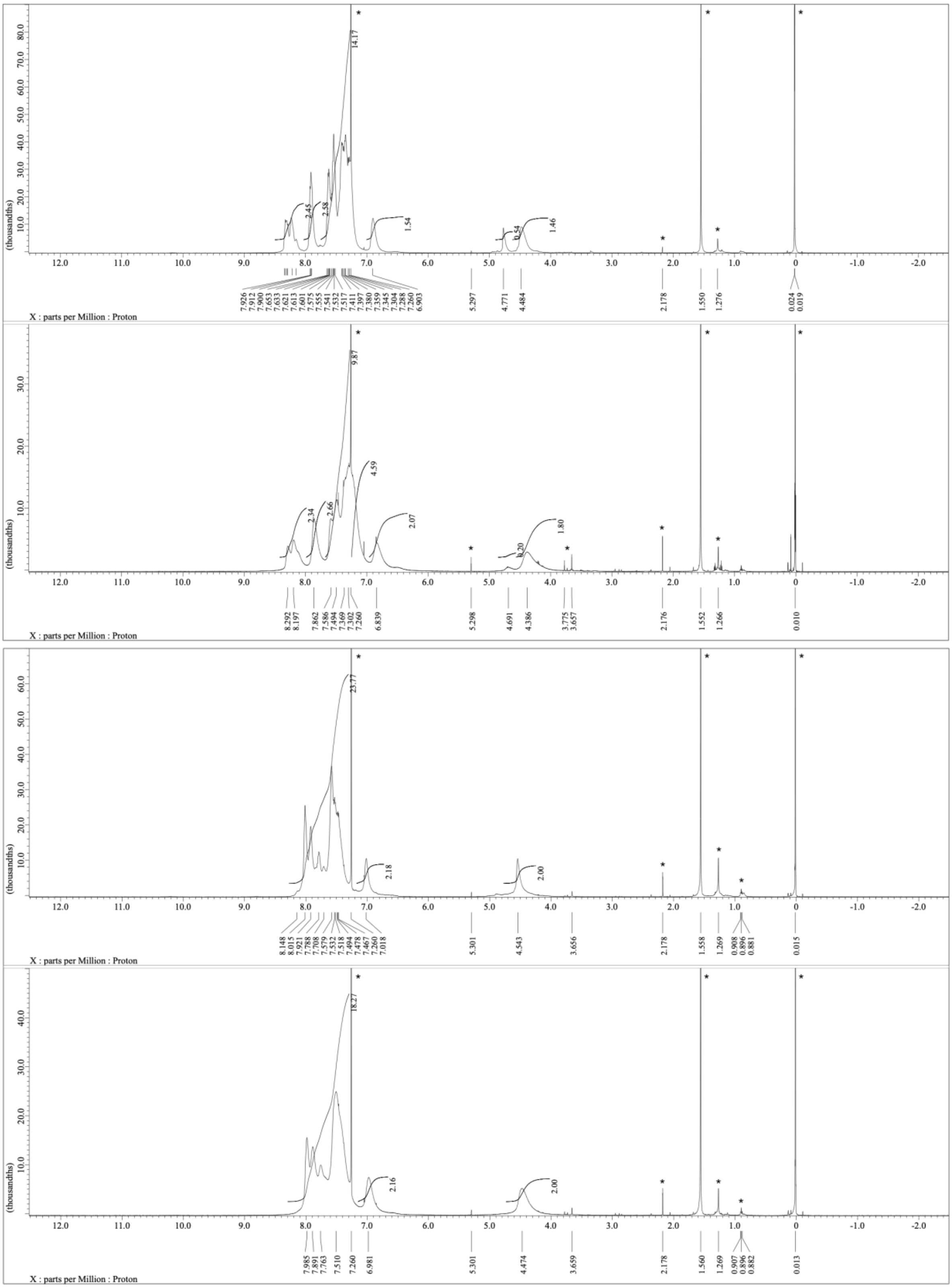

Figure S315. ^{1}H NMR spectra of a) **[4.3.3]_oligo** (top) and **[4.3.3]_linear** (bottom), and b) **[4.3.3]_oligo** (top) and **[3.3.3]_linear** (bottom) in $CDCl_3$ at room temperature. Peaks marked with * are due to residual solvents and impurities.

a)

b)

c)

d)

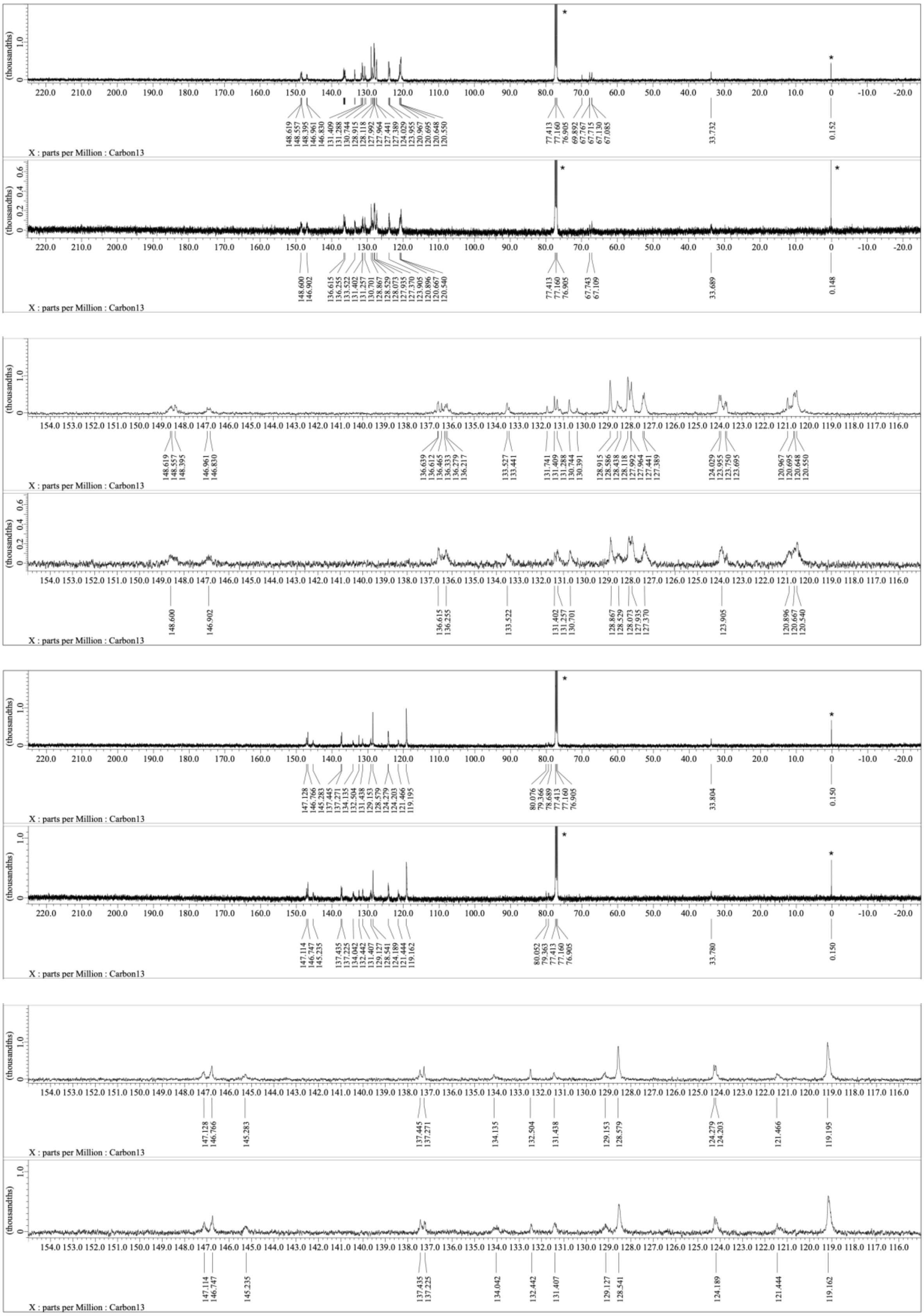

Figure S316. ^{13}C NMR spectra of a) **[4.3.3]_oligo** (top) and **[4.3.3]_linear** (bottom), and c) **[4.3.3]_oligo** (top) and **[3.3.3]_linear** (bottom) in $CDCl_3$ at room temperature, and b,d) their aromatic ranges. Peaks marked with * are due to residual solvents and impurities.

4. HR APCI–orbitrap-MS, ESI–orbitrap-MS, and EI–selector-MS

a)

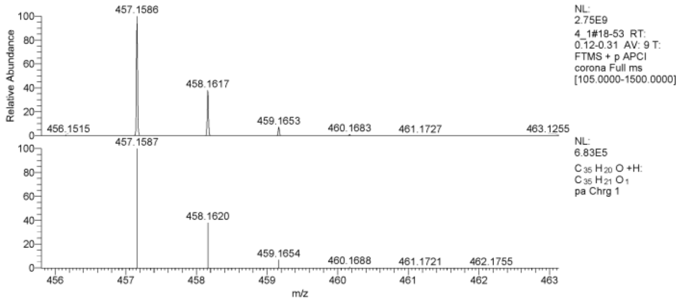

b)

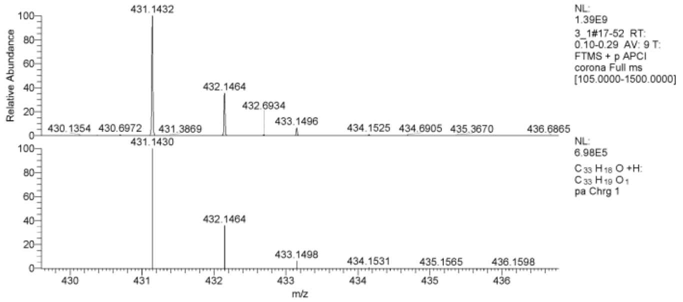

c)

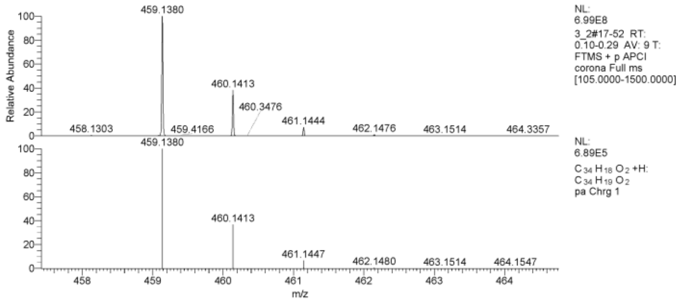

d)

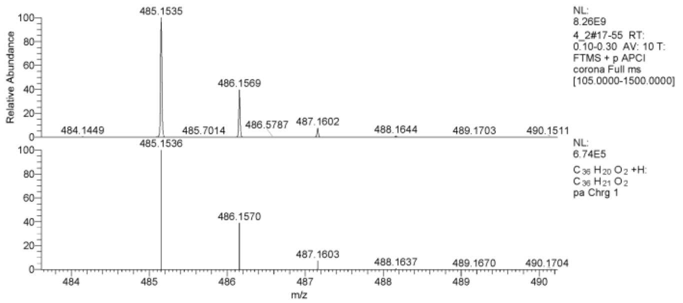

e)

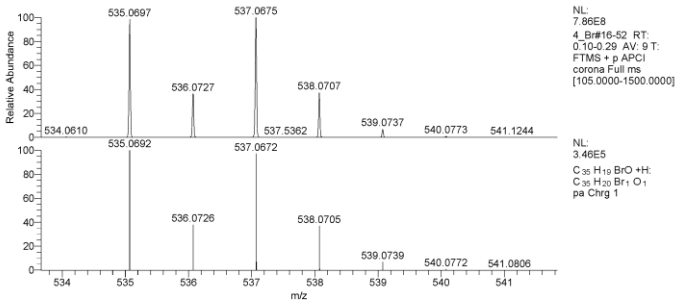

Figure S401. Observed (top) and simulated (bottom) HR-APCI–orbitrap-MS of a) **[4.3.3]_CHO**, b) **[3.3.3]_CHO**, c) **[3.3.3]_2CHO**, d) **[4.3.3]_2CHO**, and e) **[4.3.3]_Br_CHO**.

a)

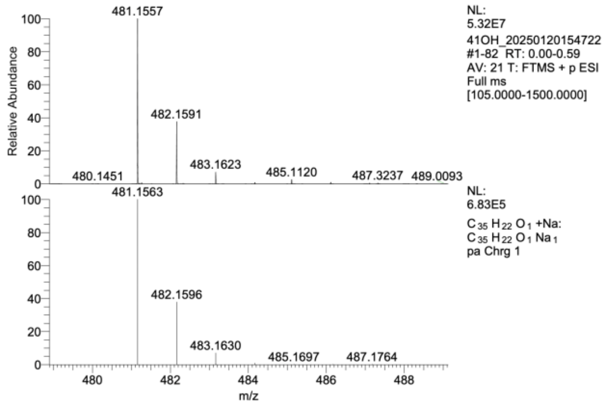

b)

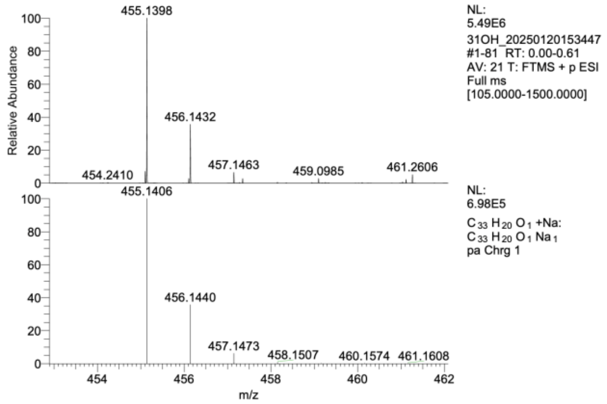

c)

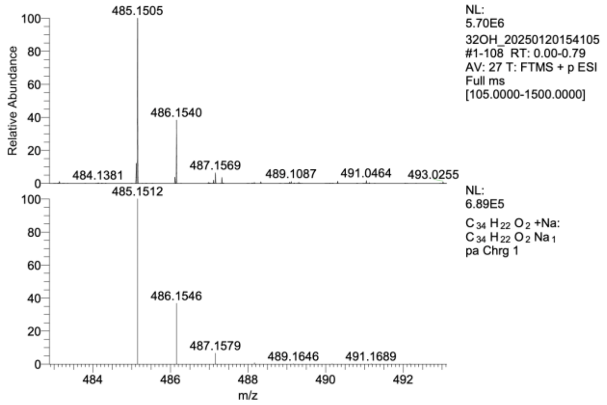

d)

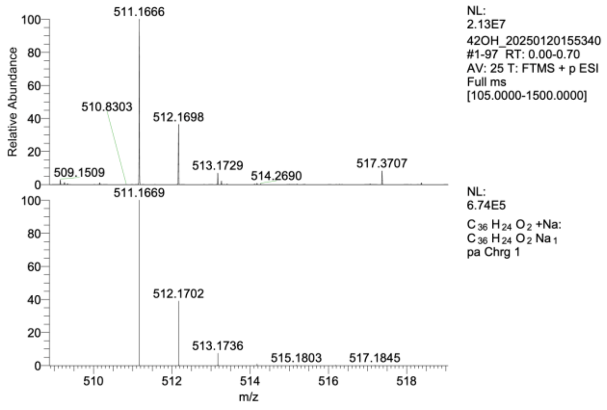

Figure S402. Observed (top) and simulated (bottom) HR-ESI–orbitrap-MS of a) **[4.3.3]_CH2OH**, b) **[3.3.3]_CH2OH**, c) **[3.3.3]_2CH2OH**, and d) **[4.3.3]_2CH2OH**.

For **[3.3.3]_CH₂OH**, **[3.3.3]_2CH₂OH**, and **[4.3.3]_2CH₂OH**, the ESI method indicated the MS peaks with lower intensities than other species, and the APCI method showed $[M-2H]^+$ or $[M-4H]^+$ signals probably owing to decomposition of the CH_2OH groups upon ionization. Therefore, the EI method was also applied.

a)

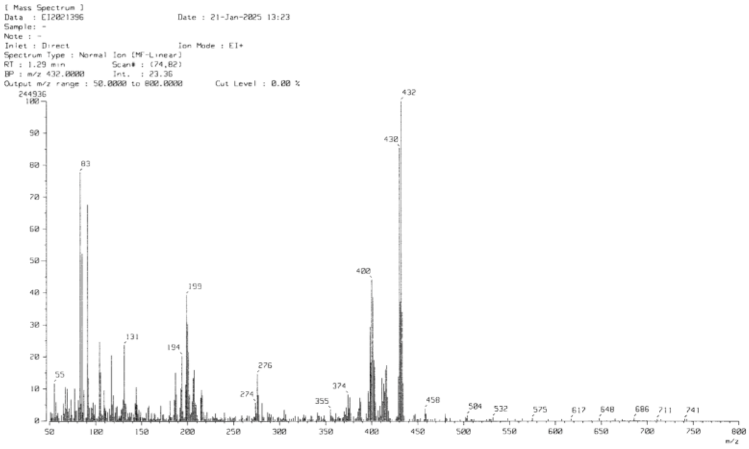

[Elemental Composition]
Data : EI2021397 Date : 21-Jan-2025 13:24
Sample: -
Note : -
Inlet : Direct Ion Mode : EI+
RT : 0.09 min Scan#: 12
Elements : C 50/0, H 60/0, O 2/0
Mass Tolerance : 5ppm, 2mmu if m/z < 400, 5mmu if m/z > 1000
Unsaturation (U.S.) : -10.0 - 40.0

Observed m/z	Int%	Err[ppm / mmu]	U.S.	Composition
432.1513	47.1	-0.3 / -0.1	24.0	C 33 H 20 O

b)

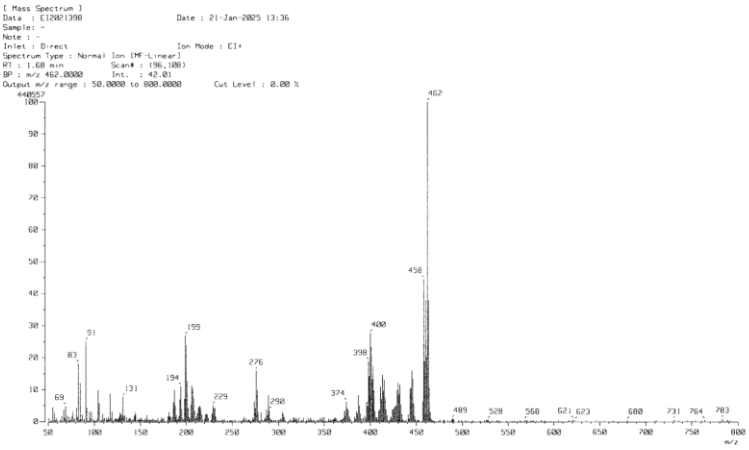

[Elemental Composition]
Data : EI2021399 Date : 21-Jan-2025 13:39
Sample: -
Note : -
Inlet : Direct Ion Mode : EI+
RT : 1.73 min Scan#: 357
Elements : C 50/0, H 60/0, O 2/0
Mass Tolerance : 5ppm, 2mmu if m/z < 400, 5mmu if m/z > 1000
Unsaturation (U.S.) : -10.0 - 40.0

Observed m/z	Int%	Err[ppm / mmu]	U.S.	Composition
462.1618	100.0	-0.4 / -0.2	24.0	C 34 H 22 O 2

c)

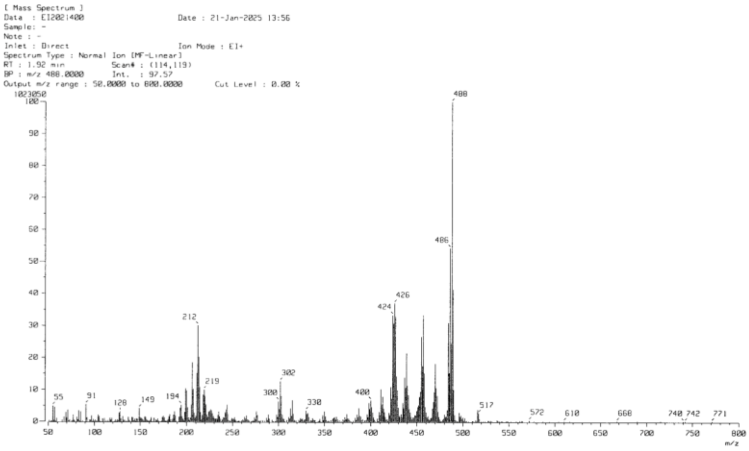

[Elemental Composition]
Data : EI2021402 Date : 21-Jan-2025 14:08
Sample: -
Note : -
Inlet : Direct Ion Mode : EI+
RT : 1.61 min Scan#: 343
Elements : C 50/0, H 60/0, O 2/0
Mass Tolerance : 5ppm, 2mmu if m/z < 400, 5mmu if m/z > 1000
Unsaturation (U.S.) : -10.0 - 40.0

Observed m/z	Int%	Err[ppm / mmu]	U.S.	Composition
488.1774	100.0	-0.4 / -0.2	25.0	C 36 H 24 O 2

Figure S403. EI–MS (left) and results for HR-EI–selector-MS (right) of a) **[3.3.3]_CH₂OH**, b) **[3.3.3]_2CH₂OH**, and c) **[4.3.3]_2CH₂OH**. Simulated spectra were not displayed because of the instrumental limitation.

5. HPLC charts

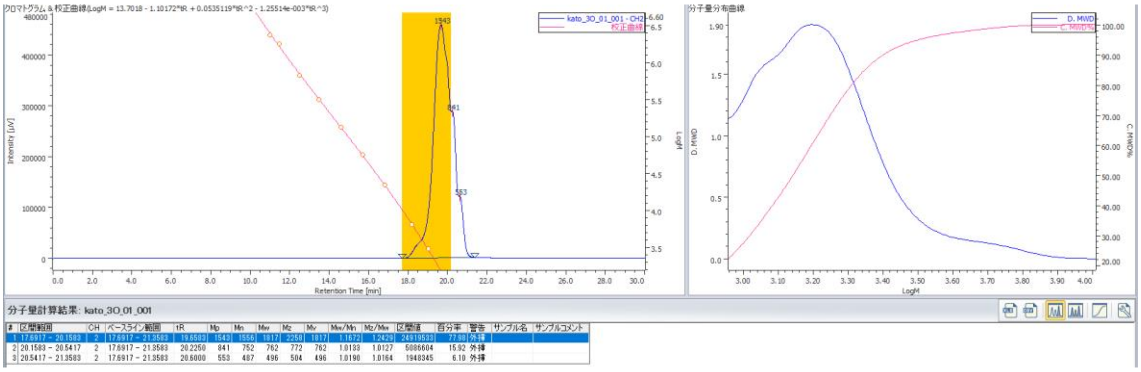

Figure S501. Copy of GPC–HPLC chart of **[3.3.3]_oligo** recorded as absorption of 300 nm light. Conditions: 25 °C; flow rate = 1.0 mL/min; eluent = THF. The chart showed that tetramer is a major component because the molecular weight of repeating unit is 414.51.

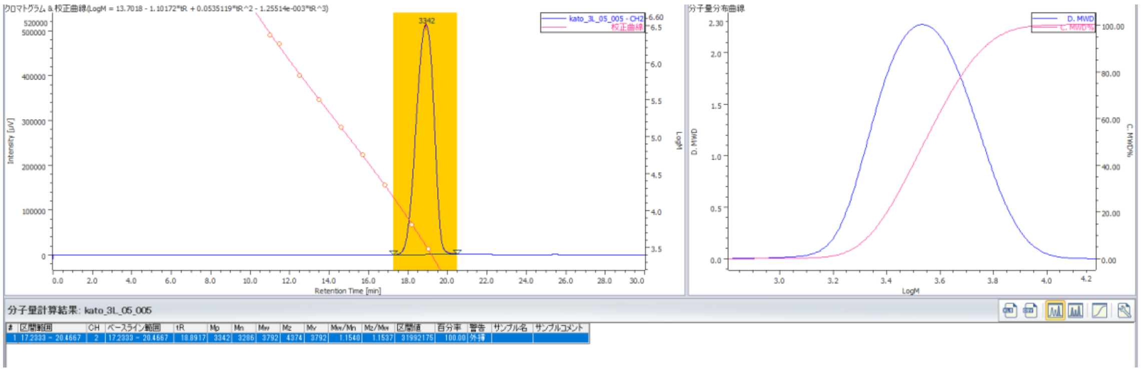

Figure S502. Copy of GPC–HPLC chart of **[3.3.3]_linear** recorded as absorption of 300 nm light. Conditions: 25 °C; flow rate = 1.0 mL/min; eluent = THF. The chart showed that octamer is a major component because the molecular weight of repeating unit is 414.51.

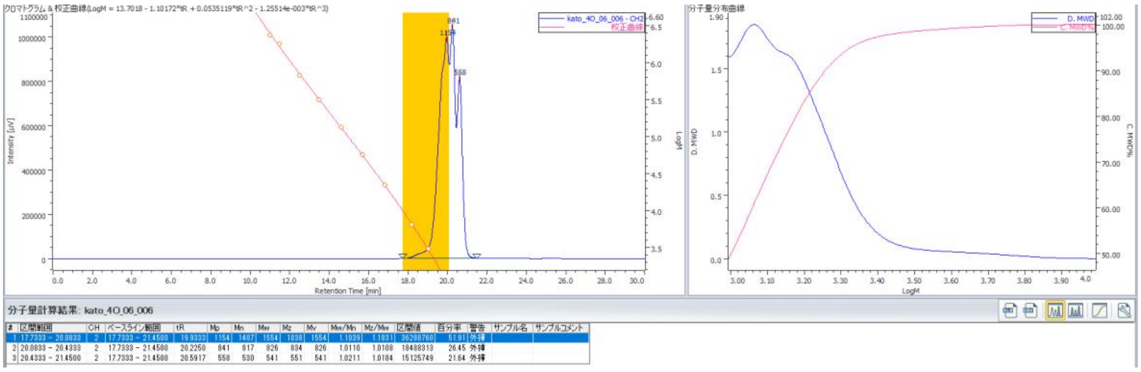

Figure S503. Copy of GPC–HPLC chart of **[4.3.3]_oligo** recorded as absorption of 300 nm light. Conditions: 25 °C; flow rate = 1.0 mL/min; eluent = THF. The chart showed that dimer and trimer are major components because the molecular weight of repeating unit is 440.55.

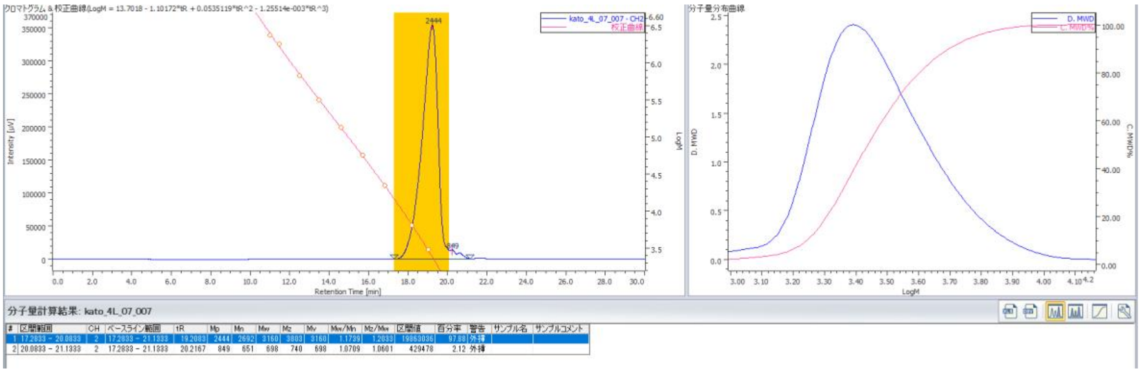

#	区間範囲	CH	ベースライン範囲	tR	Mp	Mn	Mw	Mz	Mv	Mw/Mn	Mz/Mw	区間値	百分率	警告	サンプル名	サンプルコメント
1	17.2833 - 20.0833	2	17.2833 - 21.1333	19.2083	2444	2692	3160	3803	3160	1.1739	1.2033	19863036	97.88	外挿		
2	20.0833 - 21.1333	2	17.2833 - 21.1333	20.2167	849	651	698	740	698	1.0709	1.0601	429478	2.12	外挿		

Figure S504. Copy of GPC–HPLC chart of **[4.3.3]_linear** recorded as absorption of 300 nm light. Conditions: 25 °C; flow rate = 1.0 mL/min; eluent = THF. The chart showed that pentamer and hexamer are major components because the molecular weight of repeating unit is 440.55.

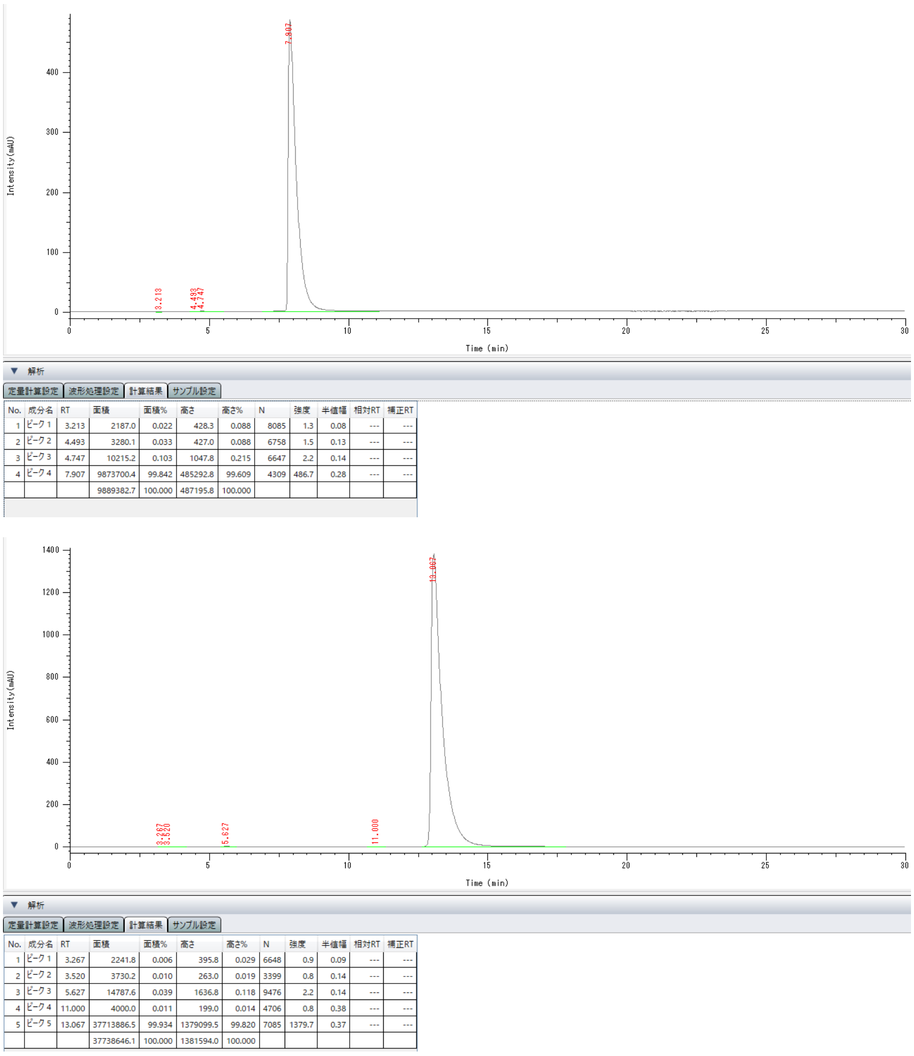

No.	成分名	RT	面積	面積%	高さ	高さ%	N	強度	半値幅	相対RT	補正RT
1	ピーク 1	3.213	2187.0	0.022	428.3	0.088	8085	1.3	0.08	---	---
2	ピーク 2	4.493	3280.1	0.033	427.0	0.088	6758	1.5	0.13	---	---
3	ピーク 3	4.747	10215.2	0.103	1047.8	0.215	6647	2.2	0.14	---	---
4	ピーク 4	7.907	9873700.4	99.842	485292.8	99.609	4309	486.7	0.28	---	---
			9889382.7	100.000	487195.8	100.000					

No.	成分名	RT	面積	面積%	高さ	高さ%	N	強度	半値幅	相対RT	補正RT
1	ピーク 1	3.267	2241.8	0.006	395.8	0.029	6648	0.9	0.09	---	---
2	ピーク 2	3.520	3730.2	0.010	263.0	0.019	3399	0.8	0.14	---	---
3	ピーク 3	5.627	14787.6	0.039	1636.8	0.118	9476	2.2	0.14	---	---
4	ピーク 4	11.000	4000.0	0.011	199.0	0.014	4706	0.8	0.38	---	---
5	ピーク 5	13.067	37713886.5	99.934	1379099.5	99.820	7085	1379.7	0.37	---	---
			37738646.1	100.000	1381594.0	100.000					

Figure S505. Copy of chiral HPLC charts of enantiopure fractions of **[4.3.3]_CH$_2$OH** recorded as absorption of 300 nm light (top: 1st fraction, bottom: 2nd fraction). Conditions: CHIRALPAK IA (Ø = 4.6 mm, *l* = 250 mm) column; room temperature; flow rate = 1.0 mL/min; eluent = CH_2Cl_2/*n*-hexane (2:1). Retention time (percentage of the peak area) was 7.91 min (99.8%) for the 1st fraction and 13.1 min (99.9%) for the 2nd one.

6. X-Ray diffraction data

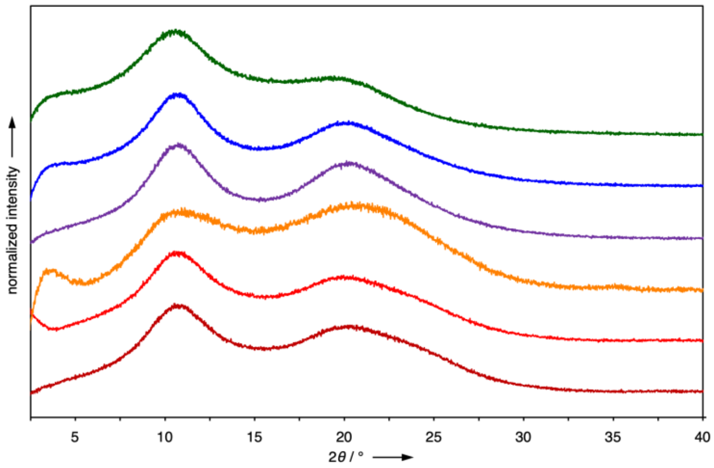

Figure S601. PXRD patterns of **[3.3.3]_oligo** (dark red), **[3.3.3]_linear** (red), **[3.3.3]_branch** (orange), **[4.3.3]_oligo** (purple), **[4.3.3]_linear** (blue), and **[4.3.3]_branch** (green).

7. DSC and TGA measurement

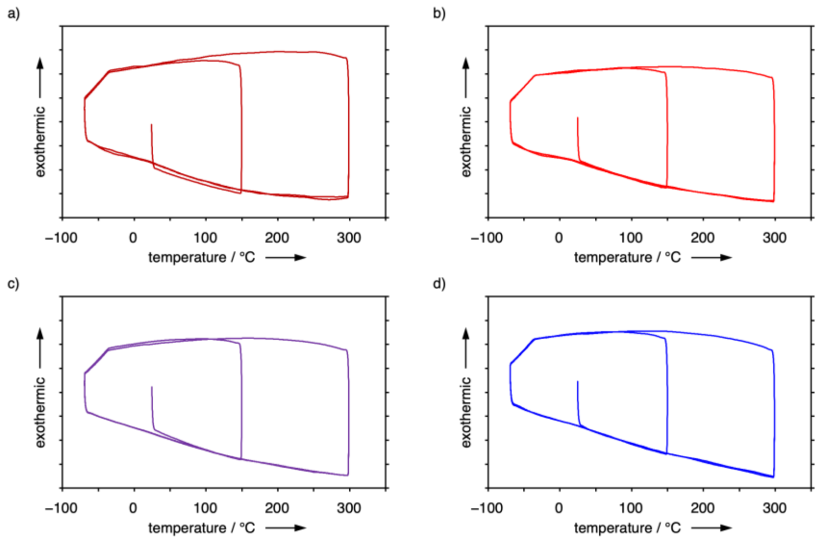

Figure S701. DSC cycling curves of a) **[3.3.3]_oligo**, b) **[3.3.3]_linear**, c) **[4.3.3]_oligo**, d) **[4.3.3]_linear** at the heating/cooling rate of 5.0 °C/min. Under nitrogen, samples were heated from 25 °C to 150 °C (1st heating) and kept at that temperature for 5 min. After cooling back to −70 °C and waiting for 30 min (1st cooling), the samples were heated to 300 °C (2nd heating). After 5 min, this cooling/heating cycle was repeated (2nd cooling/3rd heating).

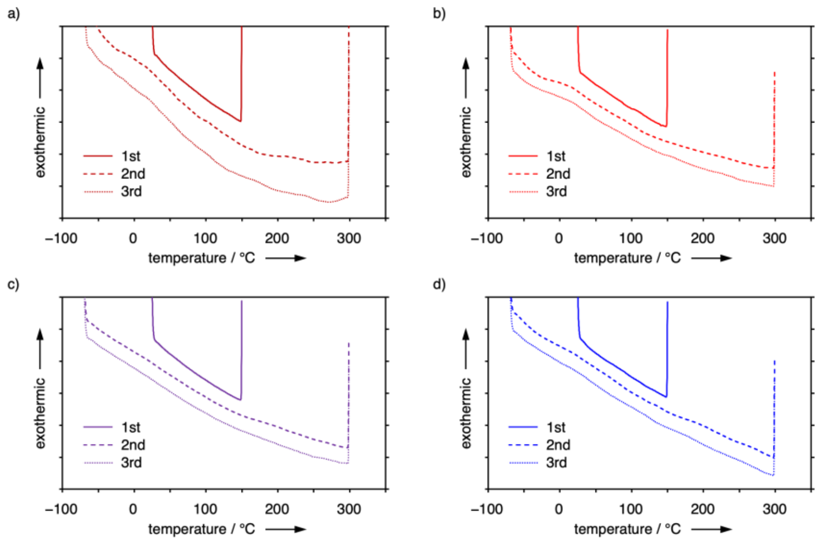

Figure S702. Stacked DSC heating curves of a) **[3.3.3]_oligo**, b) **[3.3.3]_linear**, c) **[4.3.3]_oligo**, d) **[4.3.3]_linear** at the rate of 5.0 °C/min. Under nitrogen, samples were heated from 25 °C to 150 °C (1st heating, solid lines) and kept at that temperature for 5 min. After cooling back to −70 °C and waiting for 30 min (1st cooling), the samples were heated to 300 °C (2nd heating, dashed lines). After 5 min, this cooling/heating cycle was repeated (2nd cooling/3rd heating, dotted lines).

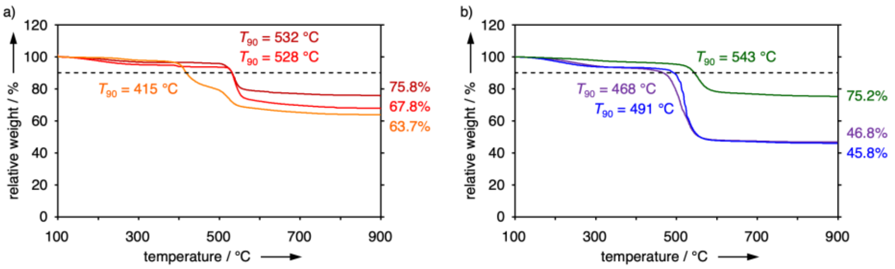

Figure S703. TGA traces of a) **[3.3.3]_oligo** (dark red), **[3.3.3]_linear** (red), **[3.3.3]_branch** (orange), and b) **[4.3.3]_oligo** (purple), **[4.3.3]_linear** (blue), and **[4.3.3]_branch** (green). Before the measurement, samples were heated at 100 °C for 60 min under nitrogen, to release the residual solvent. Then, the samples were heated from 100 °C to 900 °C at the rate of 5.0 °C/min under air.

8. Gas adsorption measurement

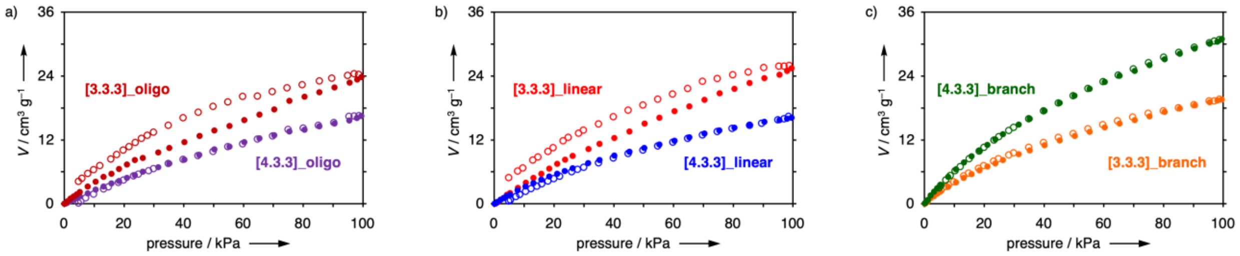

Figure S801. CO_2 gas adsorption (filled circles) and desorption (open circles) isotherms at 298 K of a) **[3.3.3]_oligo** (dark red), **[3.3.3]_linear** (red), **[3.3.3]_branch** (orange), **[4.3.3]_oligo** (purple), **[4.3.3]_linear** (blue), and **[4.3.3]_branch** (green).

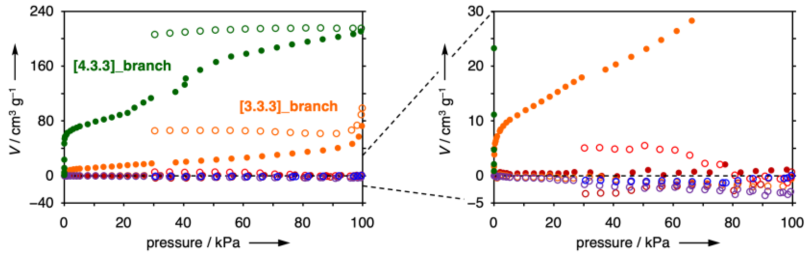

Figure S802. N_2 gas adsorption (filled circles) and desorption (open circles) isotherms at 77 K of **[3.3.3]_oligo** (dark red), **[3.3.3]_linear** (red), **[3.3.3]_branch** (orange), **[4.3.3]_oligo** (purple), **[4.3.3]_linear** (blue), and **[4.3.3]_branch** (green).

Table S801. Gas adsorption properties.[a]

	V (CO_2) [cm^3 g^{-1}]	V_m [cm^3 g^{-1}]	B [Pa^{-1}]	K_H [cm^{-3} g^{-1} Pa^{-1}]	V (N_2) [cm^3 g^{-1}]	S_{BET} [m^2 g^{-1}]
**[3.3.3]_oligo**	22	38	1.5×10	5.9×10^2	–	–
**[3.3.3]_linear**	24	55	6.8	3.7×10^2	–	–
**[3.3.3]_branch**	18	20	2.5×10	5.0×10^2	40	61
**[4.3.3]_oligo**	15	47	6.8	3.2×10^2	–	–
**[4.3.3]_linear**	15	23	1.6×10	3.6×10^2	–	–
**[4.3.3]_branch**	29	35	2.2×10	7.6×10^2	203	323

[a] V (CO_2), CO_2 uptake (STP) at 90 kPa; V_m, monolayer adsorption capacity (STP) for CO_2; B, Langmuir parameter for binding strength; K_H, Henry constant; V (N_2), N_2 uptake (STP) at 90 kPa; S_{BET}, surface area. Langmuir fitting was performed using 15 data points (2.1–40.2 kPa) for **[3.3.3]_oligo**, **[3.3.3]_linear**, **[4.3.3]_oligo**, and **[4.3.3]_linear**, and 10 data points (2.1–21.0 kPa) for **[3.3.3]_branch** and **[4.3.3]_branch**. Henry constants were calculated form the Langmuir parameters, V_m and B. BET surface area were estimated from 8 data points (10.5–29.3 kPa) for **[3.3.3]_branch** and 5 data points (10.5–21.2 kPa) for **[4.3.3]_branch**.

9. Theoretical calculations

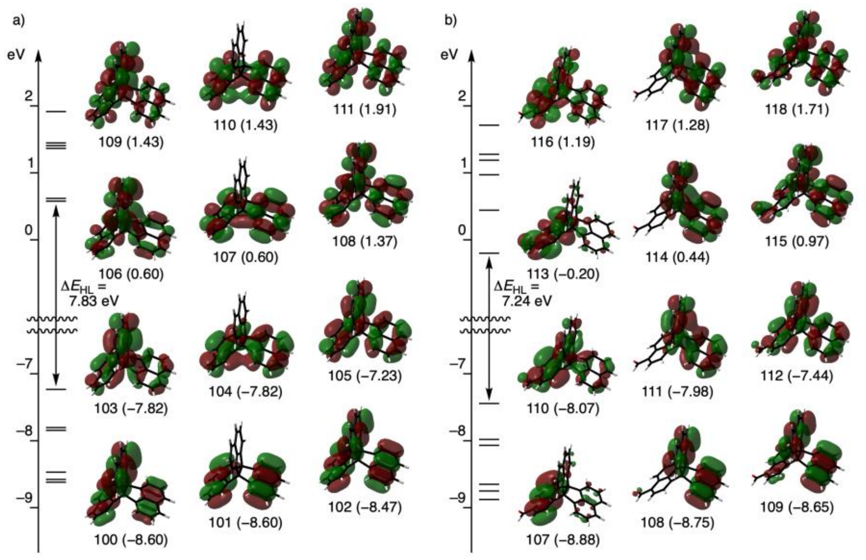

Figure S901. Energy diagrams and Kohn–Sham orbital representations of **[3.3.3]**[S2] and **[3.3.3]_CHO** (isovalue: 0.02).

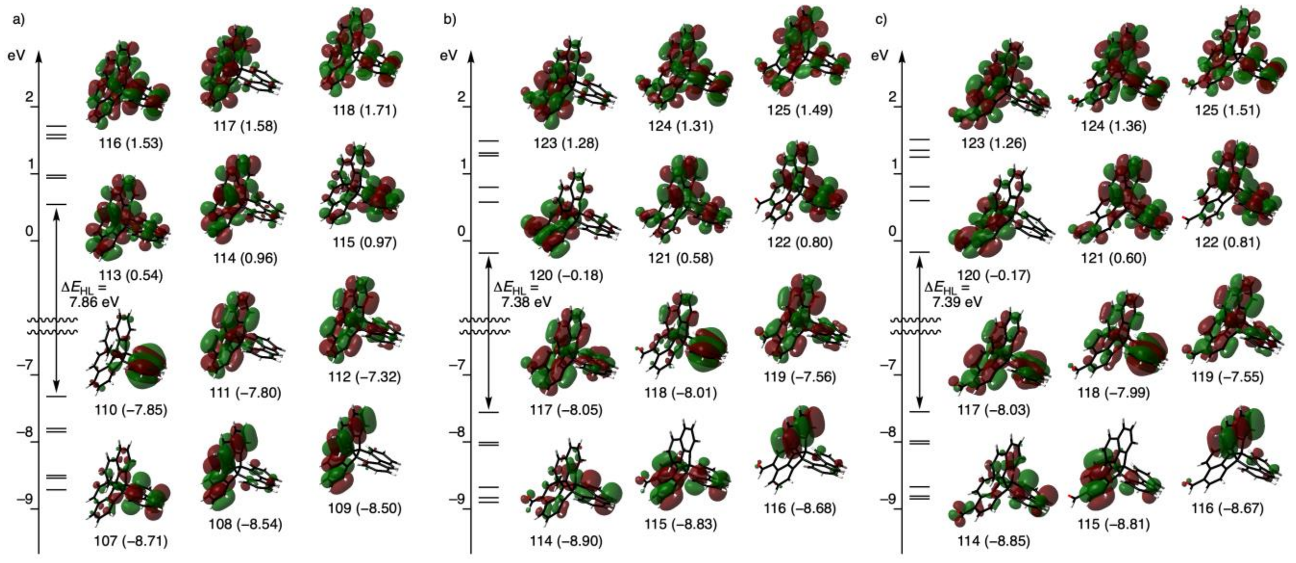

Figure S902. Energy diagrams and Kohn–Sham orbital representations of **[4.3.3]**,[S2] **[4.3.3]_CHOa**, and **[4.3.3]_CHOb** (isovalue: 0.02). Due to (*P*/*M*)-twisted conformations of the [4.3.3]propellane skeletons, **[4.3.3]_CHOa** (electronic energy, −1420.597675 Hartree) and **[4.3.3]_CHOb** (−1420.600589 Hartree) were calculated as diastereomeric states, and the latter was more stable than the former by 7.65 kJ mol^{-1}.

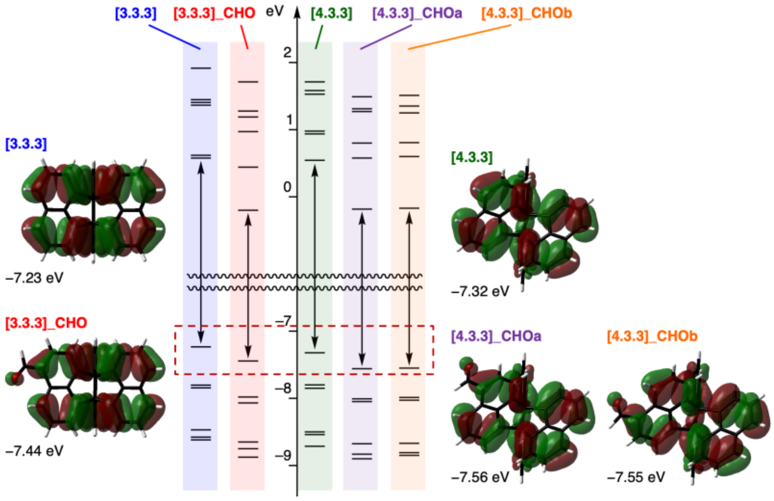

Figure S903. Comparison of energy diagrams and highest occupied molecular orbitals (HOMOs) of **[3.3.3]**,[S2] **[3.3.3]_CHO**, **[4.3.3]**,[S2] **[4.3.3]_CHOa**, and **[4.3.3]_CHOb** (isovalue: 0.02). Formylation lowered the HOMO energies by 0.21–0.24 eV but did not alter the HOMO distributions significantly.

10. References

[S1] K. Kato, S. Tanaka, N. Seto, K. Wada, M. Gon, S. Fa, S. Ohtani, K. Tanaka, T. Ogoshi, *Chem. Commun.* **2023**, *59*, 7080–7083.

[S2] K. Kato, K. Tanaka, S. Okada, T. Kaneda, S. Ohtani, T. Ogoshi, *Chem. Eur. J.* **2024**, *30*, e202402828.

[S3] T. Kubo, S. Miyazaki, T. Kodama, M. Aoba, Y. Hirao, H. Kurata, *Chem. Commun.* **2015**, *51*, 3801–3803.

[S4] Gaussian 16, Revision C.01, M. J. Frisch, G. W. Trucks, H. B. Schlegel, G. E. Scuseria, M. A. Robb, J. R. Cheeseman, G. Scalmani, V. Barone, G. A. Petersson, H. Nakatsuji, X. Li, M. Caricato, A. V. Marenich, J. Bloino, B. G. Janesko, R. Gomperts, B. Mennucci, H. P. Hratchian, J. V. Ortiz, A. F. Izmaylov, J. L. Sonnenberg, D. Williams-Young, F. Ding, F. Lipparini, F. Egidi, J. Goings, B. Peng, A. Petrone, T. Henderson, D. Ranasinghe, V. G. Zakrzewski, J. Gao, N. Rega, G. Zheng, W. Liang, M. Hada, M. Ehara, K. Toyota, R. Fukuda, J. Hasegawa, M. Ishida, T. Nakajima, Y. Honda, O. Kitao, H. Nakai, T. Vreven, K. Throssell, J. A. Montgomery, Jr., J. E. Peralta, F. Ogliaro, M. J. Bearpark, J. J. Heyd, E. N. Brothers, K. N. Kudin, V. N. Staroverov, T. A. Keith, R. Kobayashi, J. Normand, K. Raghavachari, A. P. Rendell, J. C. Burant, S. S. Iyengar, J. Tomasi, M. Cossi, J. M. Millam, M. Klene, C. Adamo, R. Cammi, J. W. Ochterski, R. L. Martin, K. Morokuma, O. Farkas, J. B. Foresman, and D. J. Fox, Gaussian, Inc., Wallingford CT, **2019**.

[S5] ϖB97X-D, long-range corrected (LC) hybrid density functional including empirical atom–atom dispersion corrections: J.-D. Chai, M. Head-Gordon, *Phys. Chem. Chem. Phys.* **2008**, *10*, 6615–6620.

[S6] K. Kato, S. Ohtani, M. Gon, K. Tanaka, T. Ogoshi, *Chem. Sci.* **2022**, *13*, 13147–13152.
